# Transgenic creeping bentgrass overexpressing *Osa‐miR393a* exhibits altered plant development and improved multiple stress tolerance

**DOI:** 10.1111/pbi.12960

**Published:** 2018-07-04

**Authors:** Junming Zhao, Shuangrong Yuan, Man Zhou, Ning Yuan, Zhigang Li, Qian Hu, Frank G. Bethea, Haibo Liu, Shigui Li, Hong Luo

**Affiliations:** ^1^ Department of Genetics and Biochemistry Clemson University Clemson SC USA; ^2^ Animal Science and Technology College Sichuan Agricultural University Chengdu Sichuan China; ^3^ College of Natural, Applied and Health Sciences Wenzhou Kean University Wenzhou Zhejiang China; ^4^ Department of Plant and Environmental Sciences Clemson University Clemson SC USA; ^5^ Rice Research Institute Sichuan Agricultural University Chengdu Sichuan China

**Keywords:** miR393, plant development, salt tolerance, drought tolerance, heat tolerance, turfgrass, transgenics, microRNA

## Abstract

MicroRNA393 (miR393) has been implicated in plant growth, development and multiple stress responses in annual species such as *Arabidopsis* and rice. However, the role of miR393 in perennial grasses remains unexplored. Creeping bentgrass (*Agrostis stolonifera* L.) is an environmentally and economically important C3 cool‐season perennial turfgrass. Understanding how miR393 functions in this representative turf species would allow the development of novel strategies in genetically engineering grass species for improved abiotic stress tolerance. We have generated and characterized transgenic creeping bentgrass plants overexpressing rice *pri‐miR393a* (*Osa‐miR393a*). We found that *Osa‐miR393a* transgenics had fewer, but longer tillers, enhanced drought stress tolerance associated with reduced stomata density and denser cuticles, improved salt stress tolerance associated with increased uptake of potassium and enhanced heat stress tolerance associated with induced expression of small heat‐shock protein in comparison with wild‐type controls. We also identified two targets of miR393, *AsAFB2* and *AsTIR1*, whose expression is repressed in transgenics. Taken together, our results revealed the distinctive roles of miR393/target module in plant development and stress responses between creeping bentgrass and other annual species, suggesting that miR393 would be a promising candidate for generating superior crop cultivars with enhanced multiple stress tolerance, thus contributing to agricultural productivity.

## Introduction

In recent years, due to the dramatic changes in climate and environment, plants are constantly challenged by a broad range of environmental stresses, which pose serious threats to crop productivity (Suzuki *et al*., 2014). Abiotic stresses such as drought, high salinity and temperature fluctuations lead to more than 50% yield loss in major crops worldwide each year (Bary *et al*., 2000; Lobell *et al*., 2011; Shao *et al*., 2009). Furthermore, abiotic stress can also adversely affect plant defence systems and increase plant susceptibility to pathogen infections (Atkinson and Urwin, 2012; Goel *et al*., 2008; Luck *et al*., 2011). Thus, plants are most likely to suffer from various environmental stresses occurring concurrently. It is estimated that almost two times of current crop production is required to meet the ever‐increasing population by 2050, whereas the arable land is not expected to expand dramatically (FAO, 1996). Therefore, it is critical to breed multiple stress‐tolerant crops.

To cope with environmental stresses, plants have evolved complex defence mechanisms involving multiple components, including pathways in signal perception and transduction, transcriptional regulation and downstream stress‐responsive gene expression (Chaves *et al*., 2003; Xiong *et al*., 2002). A large number of stress‐responsive genes that have been identified can be further classified into two classes. The first class of stress‐responsive genes encodes functional proteins that directly contribute to the protection of plant cells from stresses, such as detoxification enzyme, water channel, osmolytes, chaperones and late embryogenesis abundant (LEA) proteins. The second class includes genes for regulatory proteins such as transcription factors (TFs), protein kinases, phosphatases and other signalling molecules as well as noncoding RNAs (Shinozaki and Yamaguchi‐Shinozaki, 2007; Shinozaki *et al*., 2003; Zhou and Luo, 2013). Manipulating specific master regulatory genes in transgenic plants have been demonstrated to be effective in engineering a broad‐spectrum stress tolerance (Chen *et al*., 2014).

MicroRNAs (miRNAs) are small regulatory RNAs that act at the post‐transcriptional level to guide target mRNA cleavage or function for translation inhibition based on the complementary sequence between miRNAs and their targets (Bartel, 2004; Beauclair *et al*., 2010). An increasing number of studies on plant miRNAs have demonstrated that they are promising candidates for enhancing multiple stress tolerance in plants, for they can target multiple genes, most of which are TFs, already known to be good candidates for crop genetic engineering (Rhoades *et al*., 2002; Wang *et al*., 2016). Besides, miRNAs also regulate other stress mediators, such as protein kinases, phytohormone signalling components and reactive oxygen species (ROS) scavenging enzymes (Bian *et al*., 2012; Raghuram *et al*., 2014; Sunkar *et al*., 2006). It is known that protein kinases play critical roles in multiple stress response (Sinha *et al*., 2011). Phytohormones (e.g. auxin, cytokines, gibberellins, abscisic acid, jasmonic acid, salicylic acid, ethylene and brassinosteroids) and ROS are also vital in signal transduction when plants encounter various adverse environmental conditions (Zhou *et al*., 2016). All these indicate that miRNAs are master regulators in plant response to various environmental cues and may have great potential in crop genetic engineering for enhanced plant performance under adverse environmental conditions. Indeed, this has been demonstrated for an increasing number of miRNAs, such as miR319, miR528, miR396 and miR408 (Chen *et al*., 2015a; Ma *et al*., 2015; Yuan *et al*., 2015; Zhou *et al*., 2013). Recently, a conserved miRNA, miR393 has been identified with the potential of mediating plant response to a variety of biotic and abiotic stresses. Overexpression of miR393 caused increased antibacterial resistance in *Arabidopsis*, but reduced drought and salinity tolerance in rice (Gao *et al*., 2011; Navarro and Jones, 2006; Xia *et al*., 2012). In rice and *Arabidopsis*, the miR393 family contains two family members, miR393a and miR393b. Although the two members have the same targets and are both involved in auxin perception regulation and cadmium stress response in rice (Ding and Zhu, 2009; Si‐Ammour *et al*., 2011), they exhibit distinctive expression patterns. In rice, miR393a expressed mainly in the crown and lateral root primordial and coleoptiles tip, while miR393b expressed in the shoot apical meristem (Bian *et al*., 2012); the expression level of the miR393a changed under salinity and alkaline stresses, whereas that of the miR393b did not (Gao *et al*., 2011). In addition to stress responses, miR393 also plays a crucial role in plant growth and development, such as leaf development, root architecture, coleoptile elongation, stomatal development and flowering (Guo *et al*., 2016a; Si‐Ammour *et al*., 2011; Vidal *et al*., 2010; Xia *et al*., 2012). Studies in *Arabidopsis* and rice showed that overexpression of miR393a increased shoot and tiller numbers, because auxin signalling was repressed through down‐regulation of miR393 targets, F‐box auxin receptors (AtTIR1, AtAFB1, AtAFB2 and AtAFB3 in *Arabidopsis*; OsTIR1 and OsAFB2 in rice) (Si‐Ammour *et al*., 2011; Xia *et al*., 2012). Although the role of miR393 has been investigated in a number of plant species including switchgrass, a C4 warm‐season perennial grass species (Liu *et al*., 2017), it has not been characterized in C3 cool‐season perennial grasses. To further our knowledge about the functions of miR393, unravelling its role in plant response to multiple environmental stresses and the underlying molecular mechanisms, we used creeping bentgrass (*Agrostis stolonifera* L.), an environmentally and economically important C3 perennial turfgrass, to carry out our study.

In this study, we have generated and characterized transgenic creeping bentgrass plants overexpressing rice *pri‐miR393a* (*Osa‐miR393a*). We found that the transgenics have fewer, but longer tillers with enhanced drought, salt, and heat stress tolerance in comparison with wild‐type controls. We also identified two targets of miR393a, *AsAFB2* and *AsTIR1*, whose expression is repressed in the transgenics. Our results reveal the distinctive roles of miR393a/target module in plant development and stress responses between creeping bentgrass and other annual species and demonstrate the importance of miR393a as a promising candidate for manipulation in generating superior crop cultivars with enhanced multiple stress tolerance, thus contributing to agricultural productivity.

## Results

### miR393 responds to salt, drought, and heat stresses and auxin treatment in creeping bentgrass

Previous studies demonstrated that miR393 is involved in plant responses to drought, salt stresses and auxin treatment in annual species (Chen *et al*., 2011, 2015b; Gao *et al*., 2011; Xia *et al*., 2012). In this study, we investigated its role in perennial grass species, creeping bentgrass. To this end, we first examined the spatial expression patterns of miR393 in creeping bentgrass. Stem‐loop RT–qPCR analysis demonstrated that the mature miR393 is most abundant in leaves followed by stems, while the expression level of mature miR393 is relatively low in roots and flowers (Figure S1a). Next, we quantitatively analysed miR393 expression in response to salt, drought, and heat stresses and IAA treatment in leaves. Mature miR393 was found to be significantly induced by these abiotic stressors and auxin treatment (Figure S1b–e), in agreement with observations in *Arabidopsis* and rice (Chen *et al*., 2011, 2015b; Gao *et al*., 2011; Xia *et al*., 2012), suggesting that miR393 is also regulated by various abiotic stresses and hormone auxin in creeping bentgrass.

### Generation of transgenic creeping bentgrass overexpressing the rice miR393 gene, *Osa‐miR393a*

miR393 response to various environmental cues and auxin treatment in turfgrass prompted us to further investigate its possible involvement in determining plant adaption to abiotic stresses. To this end, a miR393 overexpression construct was prepared and introduced into creeping bentgrass ‘Penn A‐4’ via *Agrobacterium tumefaciens*‐mediated plant transformation. As shown in Figure S2a, the stem‐loop precursor of rice *Osa‐miR393a* gene was under the control of CaMV35S promoter and linked to a CaMV35S promoter‐driving hygromycin resistance gene, *Hyg*. Multiple transgenic lines were identified through *Hyg* gene and *Osa‐miR393a* amplification, respectively (Figure S2b,c). All lines showed transcription of the rice *pri‐miR393a* (see examples in Figure S2c). Interestingly, two independent transgenic lines, TG3 and TG5, displayed significantly higher *pri‐miR393a* transcription than others (Figure S2c) and exhibited extremely dwarf phenotype with early senescence and died unexpectedly (Figure S3a). It seems that the miR393‐mediated plant development may be dose‐dependent. High‐dose miR393 would significantly impact plant development, resulting in severely dwarf phenotype, and even lethal in the worst‐case scenario. Elevated transcripts of the mature *Osa‐miR393a* in transgenic lines compared to wild‐type control plants revealed by stem‐loop RT–qPCR analysis further confirm the proper processing of the primary miRNAs sequence of the rice *Osa‐miR393a* into mature miRNAs in creeping bentgrass (see examples in Figure S2d for normal type transgenics). It should be noted that contrary to that in TG2 and TG4, the accumulations of the pri‐miR393 and the mature miR393 are not positively correlated in TG1 (Figure S3c,d). This phenomenon has also previously been observed for other miRNA genes in transgenics of different plant species, for example the primary and mature miR828 in *Arabidopsis* and the primary and mature miR393 in switchgrass (Liu *et al*., 2017; Yang *et al*., 2013). Presumably, the primary transcripts of the overexpressed miRNA (pri‐miRNA) might be more efficiently processed into its mature form in some transgenic events, and therefore, the remaining pri‐miRNA becomes much less than the mature miRNA. This higher level of mature miR393 accumulation in TG1 than in TG2 and TG4 corresponds well with its slightly different phenotype from that in TG2 and TG4 as shown in Figure S3. TG1 exhibits a semi‐dwarf like phenotype compared to the other two transgenic lines with lower levels of mature miR393 (TG2 and TG4) when they are all densely grown in a big pot. The three independent lines, TG1, TG2 and TG4, were chosen as transgenic representatives for further characterization of plant development and stress response.

### *Osa‐miR393a* transgenics exhibit altered plant development

**Figure 1:**
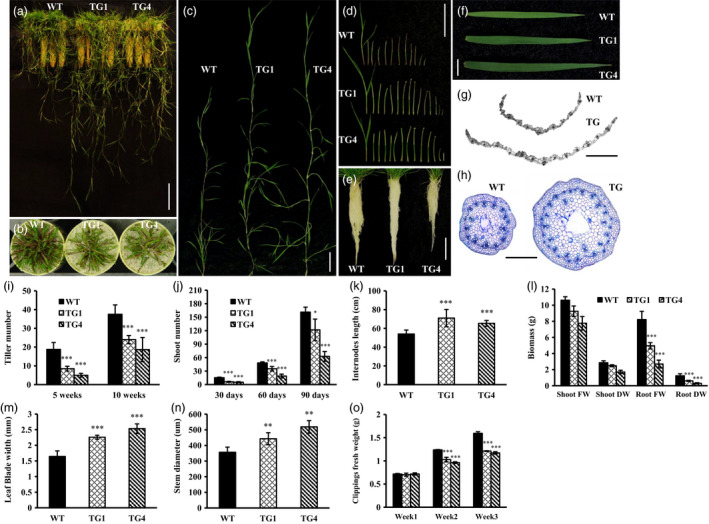
Development of wild‐type (WT) and transgenic (TG) plants. (a) Ten‐week‐old wild‐type and transgenic plants initiated from a single tiller. Bar = 10 cm. (b) Tiller number of 10‐week‐old wild‐type and transgenic plants initiated from a single tiller. (c) Close‐up of the longest tillers from wild‐type and transgenic plants. Bar = 5 cm. (d) All internodes from the representative longest tiller were sliced from top to bottom and displayed in order from left to right. Bar = 5 cm. (e) Root system of 10‐week‐old wild‐type and transgenic plants initiated from a single tiller. Bar = 5 cm. (f) Top fully developed leaf from the representative tillers of wild‐type and transgenic plants. Bar = 5 cm. (g) Cross‐sectional images of wild‐type and transgenic leaves. Bar = 200 μm. (h) Cross‐sectional images of wild‐type and transgenic stems. Bar = 200 μm. (i) Tiller number in wild‐type and transgenic plants 5 and 10 weeks after initiation from a single tiller. (j) Shoot number in wild‐type and transgenic plants 30, 60 and 90 days after initiation from a single tiller. (k) Statistical analysis of the longest tiller length between representative wild‐type and transgenic plants. (l) Statistical analysis of biomass between representative wild‐type and transgenic plants. (m) Statistical analysis of the leaf blade width between representative wild‐type and transgenic plants. (n) Statistical analysis of the stem diameter between representative wild‐type and transgenic plants. (o) Fully developed WT and TG lines (TG1, TG4) with ten tillers grown in cone‐tainers were mowed weekly to the same height. Clipping fresh weight was measured at the end of 1st, 2nd and 3rd weeks. Data are presented as means (n = at least 5), and error bars represent SD. Asterisks indicate a significant difference between the wild‐type and each transgenic line at *P <* *0.05; **P <* *0.01; and ***P <* *0.001 by Student's *t*‐test.

To determine the phenotypic impact of induced miR393 expression, wild‐type and transgenic creeping bentgrass plants initiated from a single tiller were analysed. The results showed that *Osa‐miR393a* plants displayed significantly fewer, but longer tillers than wild‐type controls (Figure 1a–c,i–k) both at the earlier (5 weeks old) and at later (10 weeks old) developmental stages (Figure 1i). We also found that transgenic plants have significantly more and longer internodes from each tiller (Figure 1d,k), wider leaves and larger stems than wild‐type controls (Figure 1f,m,n). Histological analysis of transgenic and wild‐type leaves and stems at the cellular level under microscope indicated that the numbers of vascular bundles in both leaves and stems were significantly increased in transgenics in comparison with wild‐type controls (Figure 1g,h). This might attribute to the wide leaf and large stem phenotypes observed in transgenic plants.

To study the potential impact of miR393 on plant growth, we measured shoot and root biomass of 10‐week‐old wild‐type and transgenic plants initiated from a single tiller and the weekly clipping fresh weight. Although compared to wild‐type controls, a decreased shoot biomass was observed in transgenic plants, the difference was nonsignificant (Figure 1l), suggesting that the increased tiller length in *Osa‐miR393a* plants might compensate for the loss in biomass caused by decreased tillering. The shoot weekly clipping fresh weight in transgenic plants was significantly lower than that in wild‐type controls (Figure 1o), indicating that the leaf biomass accumulation of the transgenic plants was slower than that of the control plants. However, the root biomass of *Osa‐miR393a* plants was significantly lower than that of the wild‐type controls (Figure 1l).

### Overexpression of miR393 leads to improved salt tolerance in transgenic plants

**Figure 2:**
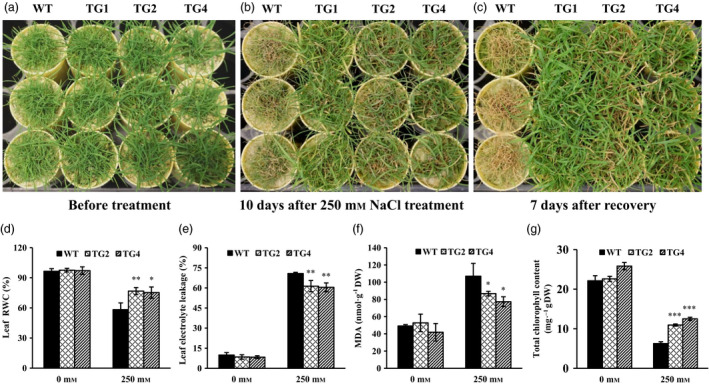
Responses of wild‐type controls (WT) and transgenics (TG) to salinity treatment. (a) Wild‐type controls and three transgenic lines initiated from the same number of tillers were fully developed in cone‐tainers for 10 weeks under normal conditions in growth room before salt stress application. (b) Performance of wild‐type and transgenic plants subjected to 250 mm NaCl treatment for 10 days. (c) Performance of wild‐type and transgenic plants 7 days after recovery from a 10‐day salt treatment. (d) Relative water contents, (e) electrolyte leakage values, (f) MDA contents and (g) total chlorophyll contents were measured before and after a 10‐day salt treatment. DW, dry weight. Data are presented as means of three biological replicates, and error bars represent SD. Asterisks indicate a significant difference between the wild‐type and each transgenic plant at *P <* *0.05; **P <* *0.01; and ***P <* *0.001 by Student's *t*‐test.

Overexpression of miR393 decreases plant salt stress resistance in transgenic rice (Xia *et al*., 2012). This observation, together with our expression analysis of miR393 in creeping bentgrass responding to salinity stress (Figure S1b), prompted us to investigate what role miR393 plays in regulating plant salt stress response in perennial grasses. As shown in Figures 2a–c and S4a–c, when subjected to 250 mm NaCl treatment, significant tissue damage and hampered plant growth were observed 10 days after salinity stress in wild‐type controls compared with transgenic plants, suggesting an improved salt tolerance in *Osa‐miR393a* transgenics.

To further elucidate the physiological mechanism of enhanced salt tolerance in *Osa‐miR393a* plants, we investigated cell membrane integrity, water status and total chlorophyll content of transgenic lines in comparison with wild‐type controls. While the rate of electrolyte leakage (EL) was similar in wild‐type controls and two transgenic lines tested under normal growth conditions, it was significantly higher in wild‐type plants than in transgenics after a 10‐d salinity stress (Figure 2e). The result indicates a better cell membrane integrity maintained in *Osa‐miR393a* transgenic lines than in wild‐type controls exposed to high salinity. Salt stress also impacts water status in plants. The relative water content (RWC), a widely used parameter to monitor plant water status was found to be significantly higher in transgenics than in wild‐type controls when subjected to salinity stress (Figure 2d), further supporting a correlation between enhanced high‐salinity resistance and elevated levels of *Osa‐miR393a* in transgenic lines. Malondialdehyde (MDA), an indicator of oxidative damage, is accumulated in plants with the development of salt stress (Halliwell and Gutteridge, 1989). We found that MDA content in wild‐type and transgenic creeping bentgrass was similar under normal growth conditions (Figure 2f). Although it was induced dramatically upon NaCl application in both *Osa‐miR393a* transgenics and wild‐type controls, the accumulation of MDA in two transgenic lines was significantly less pronounced than that in wild‐type controls (Figure 2f) and therefore less leaf damage in transgenic lines than in wild‐type controls. Moreover, during salt treatment, two transgenic lines maintained significantly higher total chlorophyll contents than wild‐type controls (Figure 2g), suggesting an improved photosynthesis in transgenics in comparison with wild‐type controls, and thereby contributing to enhanced salt stress resistance.

### Overexpression of *Osa‐miR393a* affects plant uptake of sodium and potassium

**Figure 3:**
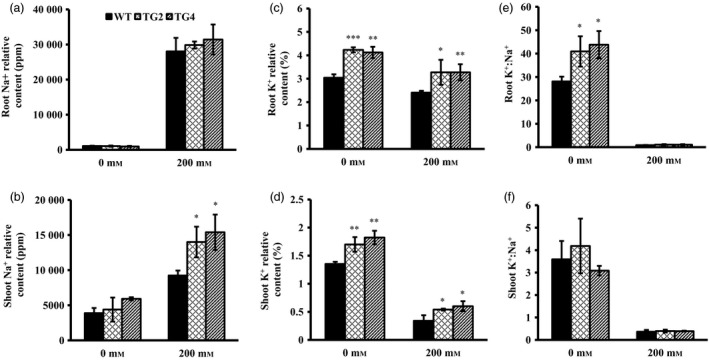
Na^+^ and K^+^ relative contents in wild‐type (WT) and transgenic (TG) plants under normal and salt stress conditions. Shoot and root tissues of the wild‐type and transgenic plants excised 10 days after NaCl treatment were used for measurement. (a) Na^+^ relative contents in root tissues of wild‐type and transgenic plants grown under normal conditions and 200 mm NaCl treatment. (b) Na^+^ relative contents in shoot tissues of wild‐type and transgenic plants grown under normal conditions and 200 mm NaCl treatment. (c) K^+^ relative contents in root tissues of wild‐type and transgenic plants grown under normal conditions and 200 mm NaCl treatment. (d) K^+^ relative contents in shoot tissues of wild‐type and transgenic plants grown under normal conditions and 200 mm NaCl treatment. (e) K^+^ : Na^+^ ratio in roots of wild‐type and transgenic plants grown under normal conditions and 200 mm NaCl treatment. (f) K^+^ : Na^+^ ratio in shoots of wild‐type and transgenic plants grown under normal conditions and 200 mm NaCl treatment. Data are presented as means of three biological replicates, and error bars represent SD. Asterisks indicate a significant difference between the wild‐type and each transgenic line at *P <* *0.05; **P <* *0.01; and ***P <* *0.001 by Student's *t*‐test.

To investigate how *Osa‐miR393a* transgenic plants perform in Na^+^ and K^+^ uptake compared to the wild‐type controls, we measured root and shoot Na^+^ and K^+^ relative contents in plants grown under normal conditions and subjected to 200 mm NaCl exposure. As shown in Figure 3a and b, no significant difference in root and shoot Na^+^ contents was observed between *Osa‐miR393a* transgenic and wild‐type plants under normal growth conditions. When exposed to salinity stress, Na^+^ accumulation in shoots and roots were increased significantly in both control and *Osa‐miR393a* plants (Figure 3a,b). More specifically, the transgenic and wild‐type plants had similar Na^+^ accumulation in roots (Figure 3a); however, Na^+^ accumulation in shoots was significantly higher in *Osa‐miR393a* transgenics than in wild‐type controls (Figure 3b). Interestingly, *Osa‐miR393a* plants accumulate more K^+^ than wild‐type controls in both roots and shoots under both normal and salinity conditions (Figure 3c,d). Upon salt treatment, although shoot and root K^+^ levels started to decline in both the *Osa‐miR393a* transgenics and wild‐type plants, the decline in wild‐type controls is more pronounced than in transgenic plants (Figure 3c,d). Under normal growth conditions, transgenic roots had significantly higher K^+^ : Na^+^ ratio than wild‐type controls due to its higher K^+^ levels (Figure 3e). Upon salt treatment, however, no significant difference in K^+^ : Na^+^ ratios in shoots and roots was detected between transgenics and wild‐type controls mostly due to the significantly acute decline of K^+^ in both genotypes (Figure 3f).

### Overexpression of miR393 improves drought tolerance in transgenic plants that is associated with reduced stomata density and denser cuticle

**Figure 4:**
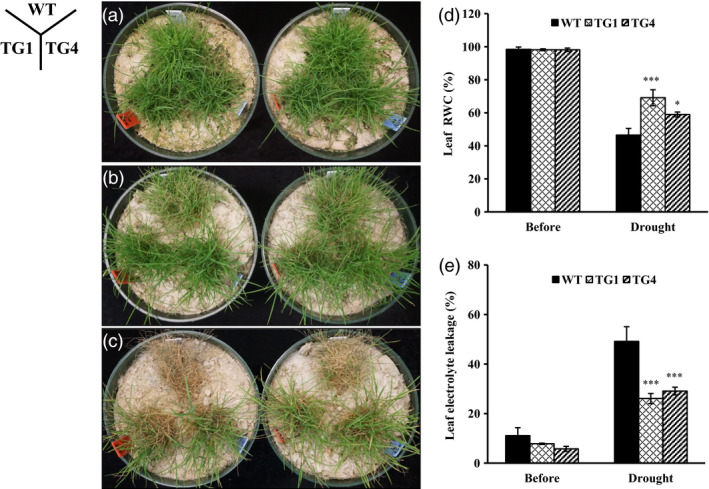
Responses of wild‐type (WT) and transgenic (TG) plants to drought stress. (a) Wild‐type controls and two transgenic lines initiated from individual tillers were fully developed in pure sand in Dillen pots for 10 weeks under normal conditions in growth room before water withholding treatment. (b) The performance of wild‐type plants and two independent transgenic lines 15 days after water withholding. (c) The performance of wild‐type plants and two independent transgenic lines 6 days after recovery. (d) Leaf relative water content (RWC) of wild‐type and transgenic plants 15 days after water withholding. (e) Leaf electrolyte leakage (EL) of wild‐type and transgenic plants 15 days after water withholding. Data are presented as means of three biological replicates, and error bars represent SD. Asterisks indicate a significant difference between the wild‐type and each transgenic line at *P <* *0.05; **P <* *0.01; and ***P <* *0.001 by Student's *t*‐test.

To study miR393 function under drought stress, mature wild‐type and *Osa‐miR393a* transgenic plants grown under optimal conditions were subjected to water withholding. Serious tissue damage was observed in wild‐type controls 15 days after water withholding, whereas most *Osa‐miR393a* transgenics remained turgid (Figure 4b). Plants were then allowed to recover by sufficient watering for 6 days. *Osa‐miR393a* transgenics were recovered from the drought‐elicited damage, whereas almost all the wild‐type controls died (Figure 4c). Measurement of plant RWC and EL revealed that water loss and drought‐elicited cell membrane damage in *Osa‐miR393a* plants were significantly less than that in wild‐type controls (Figure 4d,e).

**Figure 5:**
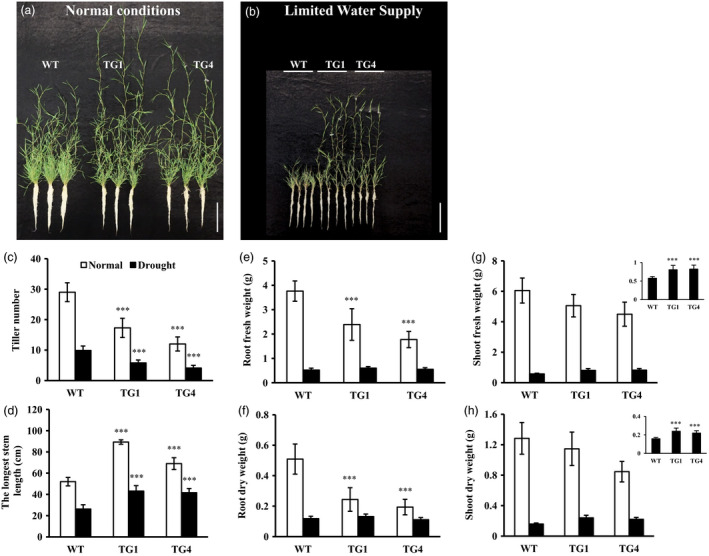
Tillering and plant development of wild‐type (WT) and transgenic (TG) plants under limited water supply for 2 months starting from a single tiller of the same size. (a) Tillering and plant growth in wild‐type and transgenic plants developed from a single tiller for 2 months (normal water supply)). Bar = 15 cm. (b) Tillering and plant growth in wild‐type and transgenic plants developed from a single tiller subjected to 2 months of drought stress (limited water supply). Bar = 15 cm. (c) Tiller numbers, (d) length of the longest stem, (e) root fresh weight, (f) root dry weight, (g) shoot fresh weight and (h) shoot dry weight of wild‐type and transgenic plants after 2 months of drought stress. Data are presented as means of five biological replicates, and error bars represent SD. Asterisks indicate significant differences between transgenic and control plants at ***P <* *0.001 by Student's *t*‐test.

Further analysis showed that leaves and stems of transgenic plants were longer than wild‐type controls when subjected to limited water supply treatment (Figure 5b,d). Although the number of *Osa‐miR393a* transgenic tiller was fewer than that of the control plants under both normal and stressed conditions, there is no significant difference in shoot biomass under normal conditions, and the shoot biomass in *Osa‐miR393a* plants was significantly increased in comparison with wild‐type controls when subjected to drought stress (Figure 5a–c,g,h). Under normal growth conditions, although transgenics have significantly fewer roots than wild‐type controls, there is no significant difference in root biomass between control and transgenic plants under limited water supply (Figure 5a,b,e,f).

**Figure 6:**
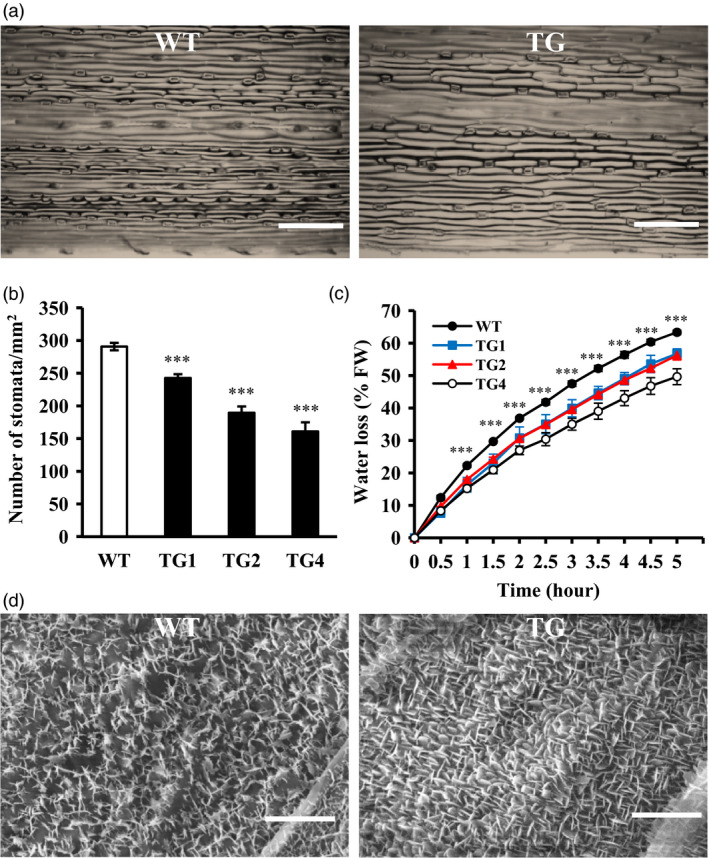
Leaf stomata and cuticle properties in wild‐type (WT) and transgenic (TG) plants. Wild‐type controls and transgenic lines initiated from a single tiller were fully developed in pure sand in Dillen pots for 5 weeks under normal conditions in growth room. (a) The epidermal cell layer of the middle leaves of wild‐type and transgenic plants under normal conditions. Bar = 250 μm. (b) Stomata density of the middle leaves of wild‐type and transgenic plants. Three random scopes were used in each repeat. (c) Water loss of wild‐type and transgenic plants under dehydration conditions. (d) SEM images of cuticle properties and wax crystals on leaf surface of wild‐type and transgenic plants under normal conditions, Bar = 2.5 μm. Data are presented as means of three biological replicates, and error bars represent SD. Asterisks indicate significant differences between transgenic and control plants at *P *<* *0.001 by Student's *t*‐test.

Wax coverage and stomata density on the leaf cuticle are correlated with water loss that impact plant tolerance to drought stress (Hepworth *et al*., 2015; Islam *et al*., 2009; Zhang *et al*., 2005). To study whether overexpression of miR393 impacts leaf surface structure in creeping bentgrass, we examined leaf stomata density and the cuticle properties of wax crystals on leaf surface with SEM in both wild‐type and *Osa‐miR393a* transgenic plants. We found that the stomata density in transgenic plants is significantly decreased compared with wild‐type controls (Figure 6a,b). Moreover, *Osa‐miR393a* plants displayed denser cuticle and lower water loss rate than wild‐type controls (Figure 6c,d). The results suggest that overexpression of miR393 improves drought tolerance in creeping bentgrass, which is associated with reduced stomata density and denser cuticle.

### Overexpression of miR393 improves heat tolerance in transgenic plants that is associated with enhanced expression of small heat‐shock protein genes

**Figure 7:**
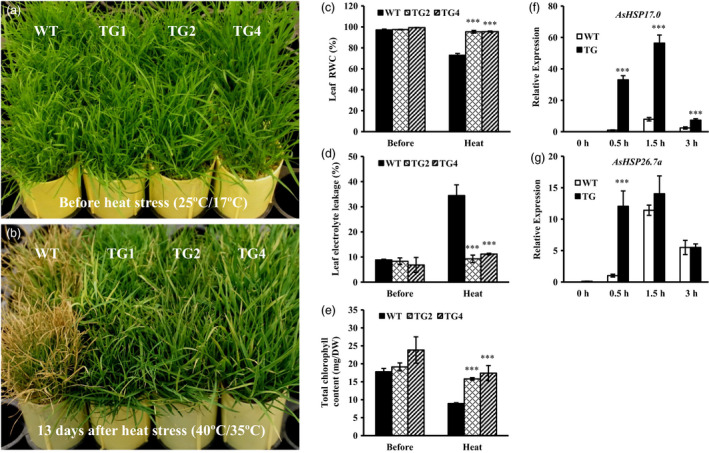
Responses of wild‐type (WT) and transgenic (TG) plants to heat stress. Wild‐type controls and three transgenic lines were fully developed in cone‐tainers for 10 weeks under normal conditions in growth room. Plants were then transferred to the growth chamber and subjected to heat stress at 40 °C in the light and 35 °C in the dark for 13 days. The relative humidity in the chamber was 60%–80%. Plant tissues were harvested for further analysis after heat treatment. (a) Wild‐type controls and three transgenic lines initiated from the same number of tillers were trimmed to the same height before the heat stress test. (b) Performance of wild‐type and transgenic plants subjected to heat stress at 40 °C in the light and 35 °C in the dark for 13 days. (c) Relative water contents (RWC), (d) electrolyte leakage (EL) values and (e) total chlorophyll contents were measured before and after 13‐day heat treatment. (f) Expression profiles of *AsHSP17.0* in wild‐type and transgenic leaf tissues under 40 °C (0–3 h). (g) Expression profiles of *AsHSP26.7a* in wild‐type and transgenic leaf tissues under 40 °C (0–3 h). DW, dry weight. Data are presented as means of three biological replicates, and error bars represent SD. Asterisks indicate a significant difference between the wild‐type and each transgenic plant at ***P <* *0.001 by Student's *t*‐test.

Considering that miR393 was induced under heat stress, we examined whether overexpression of miR393 alters plant response to high temperature. To this end, well‐developed wild‐type and *Osa‐miR393a* plants grown under optimal conditions (day 25 °C/night 17 °C) were subjected to heat treatment (day 40 °C/night 35 °C) (Figures 7 and S5). As shown in Figure 7a,b, 13 days of heat stress lead to severe leaf damage in wild‐type plants in comparison with three transgenic lines. Leaf RWC was significantly higher in transgenics than in wild‐type controls after heat treatment (Figure 7c), indicating an increased capacity of water retention in miR393 transgenic plants. The EL value was significantly decreased in transgenic lines in comparison with control plants (Figure 7d), which implies less degree of cell membrane damage in transgenic plants than in wild‐type controls. In addition, transgenic plants have higher total chlorophyll content than wild‐type controls during heat stress (Figure 7e), suggesting less leaf senescence in transgenics than in wild‐type controls. Heat stress leads to significant decline in plant biomass. We therefore also investigated the change in biomass of miR393 transgenic and wild‐type plants under heat stress. All the *Osa‐miR393a* transgenic lines displayed less reduction in shoot and root biomass than wild‐type controls (Figure S6).

Heat‐shock proteins (HSPs) are responsible for protein folding, assembly and stabilizing under normal cellular processes and refolding during stress conditions (Wang *et al*., 2004). They have previously been implicated in heat tolerance in cool‐season turfgrass (Zhan *et al*., 2011). For example, the accumulation of a small HSP, HSP25, was positively correlated with the thermotolerance in creeping bentgrass (Park *et al*., 1996). To investigate the mechanism of miR393‐mediated plant response to heat stress at molecular level, we analysed expression of *AsHSP17.0* and *AsHSP26.7a*, the two small HSP genes responding to high temperature in wild‐type and transgenic plants (Li *et al*., 2013, 2017; Sun *et al*., 2016). Quantitative RT–PCR shows that *AsHSP17.0* and *AsHSP26.7a* are induced during heat stress in both transgenic plants and controls, but the expression levels are higher in transgenics than in wild‐type controls (Figure 7f,g). The result suggests that miR393 might participate in the regulation of HSP gene expression, therefore contributing to plant protection against heat stress.

### *Osa‐miR393a* putative target identification and their responses to stresses and auxin

**Figure 8:**
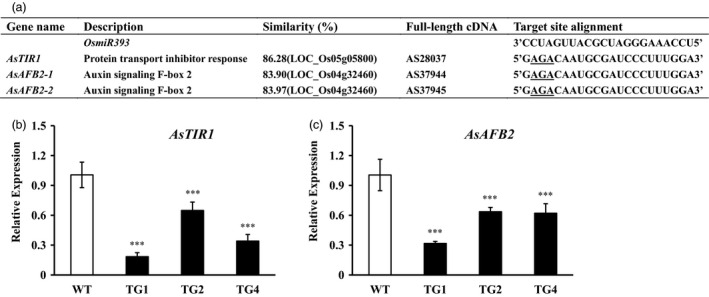
Putative miR393 target identification in creeping bentgrass. (a) Comparison of target sites in the three putative miR393 target genes in creeping bentgrass with the mature sequence of *Osa‐miR393*. Expression levels of *AsTIR1* (b) and *AsAFB2* (c) in wild‐type plants (WT) and three transgenic (TG) lines examined via RT–qPCR. Data are presented as means of three biological replicates, and error bars represent SD. Asterisks indicate a significant difference of expression levels between the wild‐type and each transgenic line at ***P <* *0.001 by Student's *t*‐test.

In rice, miR393 targets the auxin receptors, OsAFB2 and OsTIR1. To study miR393 targets in creeping bentgrass, *OsAFB2* and *OsTIR1* homologs, *AsAFB2* and *AsTIR1* were identified through blasting *OsAFB2* and *OsTIR1* against the transcriptome of creeping bentgrass generated via deep sequencing (Figure 8a, Table S2, Figures S7 and S8). Interestingly, there are two putative orthologs of rice *OsAFB2*,* AsAFB2*‐1 and *AsAFB2*‐2, in creeping bentgrass genome. *AsAFB2*‐1 and *AsAFB2*‐2 share high identity in their amino acid sequences (Figure S8). The transcripts of *AsAFB2* and *AsTIR1* in *Osa‐miR393a* transgenic lines and wild‐type controls were measured by quantitative RT–PCR analysis. We observed a reduction in the transcript levels of *AsAFB2* and *AsTIR1* in three transgenic lines in comparison with wild‐type controls (Figure 8b,c), indicating that *AsAFB2* and *AsTIR1* are negatively regulated by miR393 and are putative targets of miR393 in creeping bentgrass.

**Figure 9:**
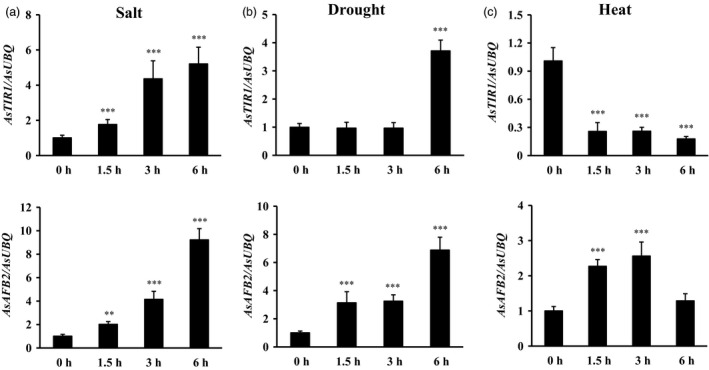
Expression patterns of the two putative miR393 targets of creeping bentgrass under salt, drought and heat stress conditions by real‐time RT–PCR analysis. (a) Real‐time RT–PCR analysis of *AsTIR1* and *AsAFB2* gene expression in wild‐type leaf tissues after exposure to 200 mm NaCl solution (0–6 h). (b) Real‐time RT–PCR analysis of *AsTIR1* and *AsAFB2* gene expression in wild‐type leaf tissues after exposure to air dry (0–6 h). (c) Real‐time RT–PCR analysis of *AsTIR1* and *AsAFB2* gene expression in wild‐type leaf tissues after exposure to heat stress at 40 °C (0–6 h). Data are presented as means of three biological replicates, and error bars represent SD. Asterisks indicate significant differences of gene expression levels between untreated and stress‐treated leaf tissues: **P <* *0.01; and ***P <* *0.001 by Student's *t*‐test.

To determine whether miR393 targets respond to abiotic stress and auxin, quantitative RT–PCR was performed with wild‐type creeping bentgrass total RNA isolated under salt, drought, heat stress and IAA treatment in leaves at various time points (0, 1.5, 3 and 6 h). The expression of *AsTIR1* increased under salt, drought stresses and IAA treatment but decreased during heat treatment (Figures 9a–c and S12). Expression levels of *AsAFB2* were induced when subjected to salt, drought and heat stresses, but decreased under IAA treatment (Figures 9a–c and S12). The observation indicates that miR393 responds to abiotic stress and auxin through regulating its targets, *AsTIR1* and *AsAFB2* in creeping bentgrass.

### Overexpression of *Osa‐miR393a* results in transcriptome changes in plant growth, development and stress regulations

**Figure 10:**
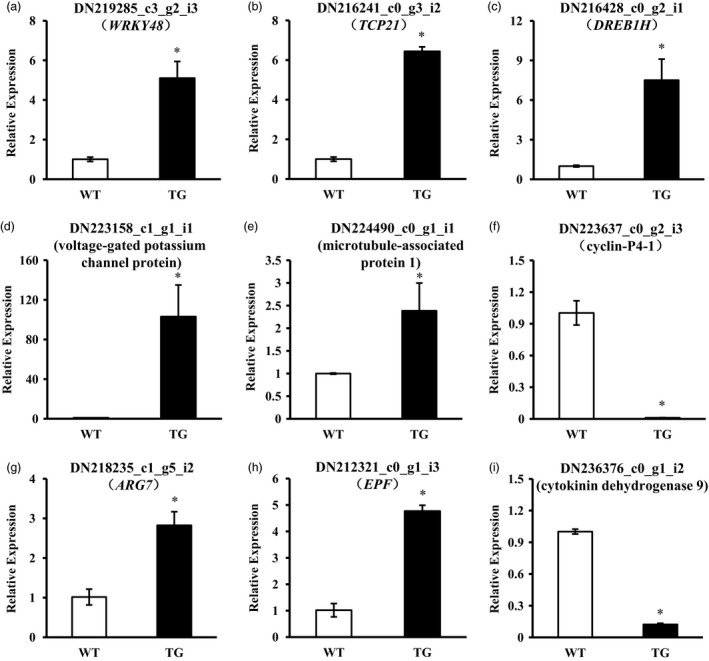
Expression levels of genes selected from RNA‐seq data in wild‐type (WT) and transgenic (TG) plants examined via RT–qPCR. Data are presented as means of three biological replicates, and error bars represent SD. Asterisks indicate a significant difference of expression levels between the wild‐type and the transgenic plants: *P <* *0.001 by Student's *t*‐test.

MiRNAs are known as master regulators in plant growth and development and response to plant stresses via coordinating multiple stress‐responsive factors. Thus, besides the direct targets of miR393, other plant growth and stress‐related elements might be regulated by miR393. To detect miR393‐mediated transcripts that may be involved in plant growth and development, a comparative transcriptome analysis was conducted using leaf samples of *Osa‐miR393a* transgenic line (TG‐4) and wild‐type (WT) controls. Under the criteria of false discovery rate <0.05 and log2 fold change ≥1, a total of 8871 unigenes were differentially expressed in *Osa‐miR393a* transgenics compared with WT controls, of which 5319 (60%) were up‐regulated, whereas 3552 (40%) down‐regulated (Figure S9). Functional annotation of putative gene products indicated that overexpression of *Osa‐miR393a* affected multiple biological processes including cellular, response to stimulus, developmental, biological regulation, transporter activity, molecular function regulator antioxidant (Figure S10). Based on the properties of miR393 and the phenotypes of *Osa‐miR393a* transgenic plants, we focused on genes involved in plant development and genes encoding transcription factors (Table 1). Fourteen predicted transcription factor genes (e.g. *WRKY*,* TCP*,* DREB*,* MADS‐box*,* MYB*) were up‐ or down‐regulated in transgenic plants, which are involved in plant development and responses to many abiotic stresses (Agarwal *et al*., 2006; Aguilar‐Martínez and Sinha, 2013; Guo *et al*., 2016a,2016b; He *et al*., 2012; Zhou *et al*., 2013). In addition, plant development‐related genes (e.g. *MAP*,* COBRA‐like*,* SAUR*,* EPF*,* CKX*) and potassium transport‐related genes were also up‐ or down‐regulated in transgenic plants. Most of these genes have previously been implicated in plant growth and development (Cockcroft *et al*., 2000; Hauser *et al*., 1995; Köllmer *et al*., 2014; Lucas *et al*., 2011; Stamm and Kumar, 2013). This implies that miR393/targets module‐mediated plant abiotic stress tolerance requires the coordination of various signalling pathways. A total of nine genes were selected for qRT–PCR analysis, including five plant growth‐related genes, one potassium transport‐related gene and three transcription factor genes (Figure 10a–i). For all the genes tested, the results obtained from two different approaches were generally in agreement with each other.

**Table 1:** Nonexhaustive list of plant development and abiotic stress response‐related genes differently regulated (*P *< 0.05) between wild‐type and *Osa‐miR393a* transgenic plants

	Gene ID	Functional annotation	log2 fold (TG/WT)
Plant development‐related genes	DN224490_c0_g1_i1	65‐kDa microtubule‐associated protein 1‐like	2.20
	DN238591_c1_g1_i2	65‐kDa microtubule‐associated protein 5‐like	−4.80
	DN234899_c3_g2_i2	65‐kDa microtubule‐associated protein 6‐like	1.84
	DN230175_c0_g1_i2	COBRA‐like protein	1.32
	DN218283_c2_g5_i3	Cyclin‐P1‐1‐like	−4.46
	DN220973_c4_g1_i4	Cyclin‐P3‐1‐like	−2.93
	DN223637_c0_g2_i3	Cyclin‐P4‐1‐like	−10.41
	DN226448_c2_g7_i1	Cyclin‐P4‐1‐like	−9.60
	DN220853_c0_g1_i3	Auxin‐responsive protein SAUR71	3.74
	DN236012_c1_g13_i1	Auxin‐responsive protein SAUR36	2.33
	DN212321_c0_g1_i3	Epidermal patterning factor‐like protein	2.42
	DN223880_c2_g3_i4	Cytokinin dehydrogenase 11	−2.19
	DN236376_c0_g1_i2	Cytokinin dehydrogenase 9	−1.91
Transcription factors	DN201921_c0_g3_i1	WRKY transcription factor 53	2.88
	DN219285_c3_g2_i3	WRKY transcription factor 48	3.34
	DN222735_c2_g1_i2	WRKY transcription factor 12	2.24
	DN223738_c3_g2_i1	WRKY transcription factor 54	−6.88
	DN230932_c5_g11_i3	WRKY transcription factor 72	−2.29
	DN214957_c5_g5_i2	Transcription factor TCP8	2.29
	DN216241_c0_g3_i2	Transcription factor TCP21	2.51
	DN223851_c1_g2_i1	Transcription factor TCP2	2.03
	DN233603_c3_g7_i2	Transcription factor TCP7	2.46
	DN204421_c0_g5_i1	Transcription factor, MADS‐box	−6.50
	DN226502_c1_g1_i1	Transcription factor, MADS‐box	2.22
	DN207922_c0_g2_i3	Transcription factor MYC/MYB	1.76
	DN224773_c1_g1_i4	Transcription factor MYC/MYB	2.12
	DN216428_c0_g2_i1	Dehydration‐responsive element‐binding protein	6.53
Potassium transport‐related genes	DN223158_c1_g1_i1	Voltage‐gated potassium channel activity	7.31
	DN232008_c2_g5_i2	Voltage‐gated potassium channel subunit beta	1.90
	DN232008_c2_g18_i1	Voltage‐gated potassium channel subunit beta	2.33

## Discussion

### MiR393*‐TIR1/AFB2* module plays an important role in regulating creeping bentgrass development and abiotic stress resistance

miR393 has previously been identified in a variety of plant species including monocots and dicots (Bian *et al*., 2012; Jagadeeswaran *et al*., 2009; Jones‐Rhoades and Bartel, 2004; Navarro and Jones, 2006; Xia *et al*., 2012; Zhang *et al*., 2009). In this study, the stem‐loop RT–qPCR analysis demonstrated that creeping bentgrass miR393 was strongly expressed in stems and leaves, but lowly expressed in roots and flowers (Figure S1a). Previous studies indicated that miR393 regulates auxin signalling pathway by negatively regulating target genes which encode F‐box auxin receptors, such as TRANSPORT INHIBITOR RESPONSE 1 (*TIR1*), AUXIN signalling F‐BOX 1, 2 and 3 (*AFB1*,* AFB2* and *AFB3*) in *Arabidopsis* and *OsTIR1* and *OsAFB2* in rice (Navarro and Jones, 2006; Si‐Ammour *et al*., 2011; Xia *et al*., 2012). Our results demonstrated that overexpression of miR393 resulted in significantly reduced expression of its predicted targets, two auxin receptor gene homologs, *AsTIR1* and *AsAFB2* in transgenic creeping bentgrass plants, suggesting that the regulation module of miR393 in the auxin pathway is conserved in different plant species. Overexpressing miR393 in transgenic rice caused reduced transcription of its targets, *OsTIR1* and *OsAFB2*, but did not change the content of IAA, which led to more tillers than wild‐type controls through hyposensitivity to the auxin signal (Xia *et al*., 2012). Interestingly, in this study, only transgenic lines (TG3 and TG5) with extremely high expression levels of *pri*‐*Osa‐miR393a* had shorter tillers than WT control plants, resulting in dwarf phenotype (Figures S2c and S3a), whereas the other transgenic lines with relatively low expression levels of *pri*‐*Osa‐miR393a* displayed fewer and longer tillers than WT controls (Figures 1a‐c,i‐k and S2c). We speculate that the dose effect of miR393 might play a delicate and interesting role in regulating its target gene expression thus impacting the overall phenotype in a way analogous to the two‐folded responses plant hormones elicit depending on their concentrations. For example, plant growth is promoted by low concentrations of auxin to a maximum, but declines with the further increase in auxin application (Foster *et al*., 1952; Marinos, 1957). Unfortunately, the dwarf transgenic lines TG3 and TG5 also exhibited an early senescence and died unexpectedly, thus preventing us from its further characterization. Tiller number is a very important turf trait and a key factor determining turf density. As shown in Figure S3, WT controls and transgenic plants were initiated from the same amount of tillers and maintained in a big pot allowing them to grow closely from each other and become quite dense, similar to that of the field‐grown turf. Under such conditions, transgenic plants display reduced growth. In particular, TG1 plants exhibit a semi‐dwarf‐like phenotype associated with a higher accumulation of the mature miR393 than TG2 and TG4. However, the transgenic plants exhibit longer internodes than WT controls initiated from a single tiller (Figure 1k). Note that under such growth conditions, the semi‐dwarf like phenotype observed in TG1 densely grown in a big pot (Figure S3) was not quite obvious (Figure 1a). This may be related to the less growth competition for each individual plant under such conditions than for the plants densely grown with more growth competition. The miR393 transgenic creeping bentgrass plants had fewer tillers than wild‐type controls, which is undesirable for turf. This important piece of information offers great insights for turfgrass genetic engineering with miRNA393, suggesting that engineering‐specific miRNA targets would be a better strategy in turf trait modification to avoid pleiotropic effects bestowed by the master regulator, miRNAs.

The role of miR393*‐TIR1/AFBs* module in regulating abiotic stress response has been well characterized in some plant species. In *Arabidopsis*, transgenic plants overexpressing miR393 displayed decreased susceptibility to auxin and enhanced plant sensitivity to salt and osmotic stress through suppressing target genes (Chen *et al*., 2011, 2015b). Similarly, overexpression of miR393 also led to decreased sensitivity to auxin and conferred reduced tolerance to salt and drought in transgenic rice due to the down‐regulation of its target mRNAs (Xia *et al*., 2012). However, unlike *Arabidopsis* and rice, transgenic tobacco overexpressing an *Arabidopsis* miR393 gene exhibited altered auxin sensitivity and enhanced salt tolerance due to auxin signal suppression via negative regulation of target *NtTIR1* (Feng *et al*., 2010). Similarly, our results clearly demonstrate that transgenic creeping bentgrass overexpressing *Osa‐miR393a* gene had enhanced drought and salt stress tolerance in comparison with wild‐type controls (Figures 2, 4 and 5). Our data together with previous research suggest that the mechanisms in which miR393*‐TIR1/AFBs* regulatory module and other related regulatory networks exert their functions to confer plant abiotic stress resistance vary between species. MiR393 expression level might also contribute to the differences in plant resistance to abiotic stresses.

F‐box protein TIR1 and AFBs function as auxin receptors to promote the ubiquitination and 26S proteasome‐mediated degradation of Aux/IAA repressors in response to auxin signals, which is necessary for auxin‐induced plant growth and development (Dharmasiri *et al*., 2005). Auxin is a vital hormone that is not only associated with the modulation of plant growth and development but also the regulation of plant resistance to abiotic stress through direct physical interaction with TIR1/AFB receptor proteins (Ding *et al*., 2008; Shibasaki *et al*., 2009). The *TIR/AFBs* play a key role in response to abiotic stress in *Arabidopsis*. *tir1afb2* and *tir1afb3* double mutants displayed enhanced tolerance to oxidative stress, and *tir1afb2* double mutant resulted in increased resistance to salinity (Iglesias *et al*., 2010). Moreover, overexpression of a miR393‐resistant form of *TIR1* (*mTIR1*) in *Arabidopsis* enhanced tolerance to salt stress resulting from increased osmoregulation and Na^+^ exclusion through auxin‐mediated downstream pathways (Chen *et al*., 2015b). In our study, *AsTIR1* and *AsAFB2* expressions are induced by many abiotic stresses, such as salt, dehydration and heat (Figure 9), suggesting that the putative miR393 target genes are directly involved in miR393‐mediated plant responses to abiotic stress. Additionally, our RNA‐seq data show that several stress‐responsive transcription factors were also up‐ or down‐regulated in *Osa‐miR393a* transgenic plants (Table 1). Thus, we speculate that *miR393* may serve as a master regulator to cope with different environmental stresses through integrating various regulatory pathways in creeping bentgrass.

### Morphological change and drought stress tolerance in *Osa‐miR393a* transgenics

Plants can cope with water deficit through morphological changes in root and leaf (Fang and Xiong, 2015). Leaves are not only very important in plant photosynthesis and respiration, but also considered to be vital in regulating stress tolerance (Atkin *et al*., 2000; Nautiyal *et al*., 2008; Terry and Ulrich, 1974). In our study, *Osa‐miR393a* transgenic plants exhibit decreased stomata density, denser cuticle on leaf surface (Figure 6a,b,d) and wider leaves (Figure 1f,g). As is known, the rate of transpiration can be regulated by stomata density and stomata opening (Mehri *et al*., 2009). Thus, decreased stomata density on leaf surface had a significant positive correlation with plant drought stress tolerance due to a reduced transpiration rate (Hepworth *et al*., 2015; Luo *et al*., 2013). As for leaf surfaces, they are covered with a cuticular wax layer that is the first barrier to protect plants against the environmental stimulation. They can effectively reduce water transpiration loss and enhance plant resistance to drought stress (Cameron *et al*., 2006, 2006; Islam *et al*., 2009; Zhang *et al*., 2005). Hence, the phenotype of denser cuticles on transgenic leaf surface is considered as a positive trait in regulating plant drought stress tolerance. However, wider leaves are actually considered as a negative trait in regulating drought stress tolerance due to increased transpiration area (Craw *et al*., 2004; Deák *et al*., 2011). Conclusively, whether the plant cultivars are more resistant to drought stress or not is dependent on the balance of trade‐offs of several morphological traits and other factors. This is similar to the case of miR319, in which other characteristics of leaves, such as stomatal aperture, stomatal density, increased leaf thickness and increased wax content on leaf surface, may compensate the water loss by wider leaves (Zhou *et al*., 2013). To further explore what caused the morphological change in *Osa‐miR319a* plants, we examined the RNA‐seq data backed up by qRT–PCR confirmation and found that the expression of *AsEPF*, a homolog of *Arabidopsis EPIDERMAL PATTERING FACTOR (EPF)*‐like gene was significantly higher in transgenics under normal conditions than that in WT controls (Figure 10h). *EPF1* and *EPF2* have been shown to negatively regulate stomatal density in *Arabidopsis* (Hara *et al*., 2007, 2009), and overexpression of a poplar *PdEPF2* leads to enhanced drought tolerance by modulating stomatal density in transgenic *Arabidopsis* (Liu *et al*., 2016). Thus, further exploration about how miR393 functions to regulate *AsEPF* expression to control stomata density change will provide information for a better understanding of molecular mechanisms of miR393‐mediated drought tolerance in transgenic creeping bentgrass.

### Mechanisms of high‐salinity resistance in *Osa‐miR393a* transgenic plant

To further explore the underlying mechanisms of the enhanced salt stress tolerance in *Osa‐miR393a* transgenic plants, we analysed Na^+^ and K^+^ uptake in both *Osa‐miR393a* transgenic and wild‐type control plants. As shown in Figure 3b,e, *Osa‐miR393a* transgenic plants perform better than wild‐type controls in maintaining K^+^ levels in shoots and roots under both the normal and stressed conditions. This phenotype correlates well with our RNA‐seq and qRT–PCR data that the expression of *AsNHX1* is significantly up‐regulated in transgenic plants (Figure S11a). Considering that *NHX1* and *NHX2* play important roles in controlling potassium uptake into vacuoles to mediate cell turgor and stomata functions in *Arabidopsis* (Barragán *et al*., 2012), these results together indicate that the impact of miR393 on potassium uptake and transport is significant. However, as for Na^+^ accumulation shown in Figure 4a,d, miR393 has no impact on roots. Interestingly, when exposed to 200 mm NaCl, *Osa‐miR393a* transgenic plants accumulate more sodium than wild‐type controls (Figure 3d). These data suggest that *Osa‐miR393a* transgenic plants do not adopt a salt exclusion mechanism to reduce sodium toxicity like what *Osa‐miR319a* plants do (Zhou *et al*., 2013). Instead, they might gain more salt tolerance through sodium sequestration into vacuoles. Thus, the increased accumulation of sodium in *Osa‐miR393a* plants could lead to decreased cell osmotic potential, thus facilitating plant water uptake and leaf turgor maintenance under salinity conditions, consistent with the observation in transgenic creeping bentgrass overexpressing an *Arabidopsis* vacuolar H^+^‐pyrophosphatase (*AVP1*) gene (Li *et al*., 2010). Indeed, the expression of *AsVP1* is significantly up‐regulated in *Osa‐miR393a* transgenic plants (Figure S11b). Although our data demonstrate the correlation between miR393 and the control of vacuolar dynamics, it remains unclear how miR393 functions in this process. It would be very interesting to dissect the role miR393 plays in determining ion exchange in creeping bentgrass by regulating *AsVP1* and *AsNHX1* in the future.

### miR393 positively regulates plant heat stress response in creeping bentgrass

Previous studies in various plant species suggested that miR393 plays a crucial role in response to multiple environmental cues, such as drought, salt, alkali, cold and aluminium stress (Bai *et al*., 2017; Gao *et al*., 2011; Liu *et al*., 2017; Xia *et al*., 2012; Xie *et al*., 2007; Zhou *et al*., 2008). However, there has been no report about the possible involvement of miR393 in plant response to heat stress so far. In this study, we show that under high temperature, *Osa‐miR393a* transgenic plants remained green with only minor heat damage and slightly reduced biomass compared to wild‐type controls, suggesting an enhanced heat tolerance in *Osa‐miR393a* transgenic plants (Figure 7). It is worth noting that TG1 seems to suffer a little more heat damage than other transgenic lines despite its enhanced heat tolerance compared to wild‐type controls (Figure S5). This might be associated with the higher expression of miR393 in this transgenic line than in the other two lines (TG2 and TG4). Interestingly, this minor difference in heat tolerance does not seem to be manifested when testing plant heat response under densely grown conditions. Presumably, the densely grown plants might be more advantageous than individually grown plants in buffering heat‐elicited damage. In Chinese cabbages, the high content of cuticular wax in heat‐tolerant cultivar plays an important role in affecting water balance in plants under heat stress, suggesting that the effects of heat stress are likely involved in drought stress under high‐temperature conditions (Kuo *et al*., 1988). Our research shows that *Osa‐miR393a* transgenic plants exhibit denser cuticle than WT controls under normal conditions (Figure 6d), which also implies higher wax content in *Osa‐miR393a* transgenic plants. Thus, we speculate that the enhanced heat stress tolerance in transgenic creeping bentgrass is associated with increased cuticular wax coverage, which can positively regulate water balance in plants under high temperature.

The heat‐shock proteins (HSPs), including small heat‐shock proteins (sHSPs), accumulate in response to heat stress. They have a chaperone activity for a protective effect at high temperature to prevent protein misfolding and aggregation (Vierling, 2003; Wang *et al*., 2004). Heat tolerance is associated with induced expression of heat‐shock proteins in plants (Queitsch *et al*., 2000; Sun *et al*., 2002; Zhong *et al*., 2013). Overexpression of HSP leads to enhanced heat tolerance has also been observed in various plant species (Kim *et al*., 2012; Merino and Gómez, 2014; Mu *et al*., 2013; Murakami *et al*., 2004; Sato and Yokoya, 2008; Sun *et al*., 2012). In creeping bentgrass, chloroplast‐localized small heat‐shock proteins (CP‐sHSPs) play an important role in heat tolerance (Wang and Luthe, 2003). It has previously been demonstrated that transgenic creeping bentgrass overexpressing *OsSIZ1*, a rice gene encoding SUMO E3 ligase, exhibit enhanced heat tolerance, and this enhancement is correlated with significantly induced expression of *ApHSP16.5* (renamed as *AsHSP17* in this study) and *AsHSP26.7a* compared to wild‐type control plants (Li *et al*., 2013). In this study, when subjected to heat stress, *AsHSP17* and *AsHSP26.7a* genes were all induced in both wild‐type and *Osa‐miR393a* transgenic plants, but the induced expression of *AsHSP17* and *AsHSP26.7a* in *Osa‐miR393a* transgenic plants was significantly more pronounced than in wild‐type controls either at all the time (*AsHSP17*) or 0.5 h after exposure to heat stress (*AsHSP26.7a*) (Figure 7f,g). This result suggests that miR393 may be involved in heat stress tolerance in creeping bentgrass via positive regulation of certain CP‐sHSPs in heat response pathways. Additionally, down‐regulated expression of the putative miR393 target genes, *AsTIR1* and *AsAFB2* in response to heat stress (Figure 9c), suggests their possible involvement in determining plant capacity of coping with heat stress. Further studies will provide information for a better understanding of the molecular mechanisms of miR393‐mediated plant resistance to heat stress through overexpression and knockout of miR393 target genes in creeping bentgrass.

**Figure 11:**
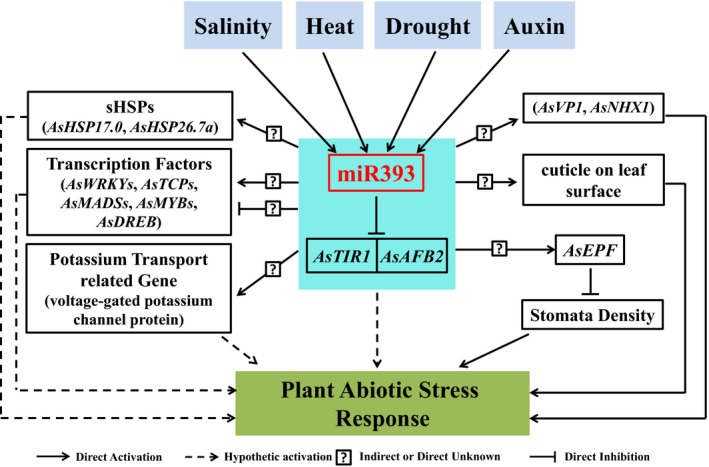
Hypothetical model of the molecular mechanisms of miR393‐mediated plant abiotic stress response in creeping bentgrass. miR393‐mediated plant abiotic stress responses may entail different mechanisms including directly repressing the expression of its targets *AsTIR1* and *AsAFB2*, positively or negatively regulating expression of *sHSPs*, transcription factor genes, potassium transport‐related gene and epidermal patterning factor gene *AsEPF* to integrate various signals in response to plant abiotic stress.

In summary, miR393 is induced during salinity, heat, drought stress and auxin treatment. miR393 mediates plant abiotic stress responses through directly repressing the expression of its targets *AsTIR1* and *AsAFB2*. In addition, miR393 positively or negatively regulates sHSPs (*AsHSP17.0* and *AsHSP26.7a*), certain transcription factors, ion transport‐related genes (*AsVP1* and *AsNHX1*) and *AsEPF*, which leads to the enhanced tolerance to salinity, heat and drought stress. Taken together, our data suggest that miR393 forms a regulatory network to integrate various signals in plant response to abiotic stress (Figure 11). Our data also demonstrate a striking difference in miR393‐mediated plant development and stress responses between various species (Liu *et al*., 2017; Xia *et al*., 2012), highlighting the significance of the species‐dependent miR393 regulatory networks. The phenotype difference between species in miR393‐mediated plant development and stress responses might result from the evolution of miRNA regulatory programs. In some plant species, conserved miRNAs may have gained unique targets over time, or alternatively, conserved miRNA‐target gene modules could have gained new functions during evolution (Willmann and Poethig, 2007). It is therefore conceivable that creeping bentgrass miR393 might gain new target and/or new miR393‐target signalling pathway, attributable to the characteristic phenotypes, such as reduced tillers and enhanced stress tolerance when overexpressing miR393 in transgenics (Figure 1). In fact, the phenomenon that conserved miRNAs play opposite roles in different plant species has previously been observed. For instance, constitutive expression of miR169 enhanced drought tolerance in transgenic tomato, but resulted in increased sensitivity to drought stress in transgenic *Arabidopsis* (Li *et al*., 2008; Zhang *et al*., 2011). Besides, the differences in plant development (such as tiller numbers) between transgenics of various plant species overexpressing miR393 might also result from varying sensitivities to hormone auxin. It is known that higher concentration of auxin promotes tiller elongation and inhibits the growth of lateral buds (apical dominance), whereas lower concentration of auxin promotes the growth of lateral tillers. Although it has been shown that miR393 overexpression leads to hyposensitivity to auxin (Chen *et al*., 2011; Liu *et al*., 2017; Xia *et al*., 2012), this sensitivity to auxin could vary among plant species, or even plant tissues. Without the knowledge about auxin concentration for optimal plant growth in a given species, it is hard to determine whether the hyposensitivity leads to the maintenance or removal of the apical dominance. Therefore, different plant species may exhibit different phenotypes under similar auxin concentration. As the upstream regulators, miRNAs not only directly affect their targets but also indirectly impact other nontarget genes. Thus, besides the direct miR393 targets, plant development and stress responses may also depend on other downstream functional genes (indirect miR393 targets), which could vary in different species, and therefore contribute differently to plant development and stress responses. Information about the species‐associated differences in miR393‐mediated plant development and stress responses greatly enriches our knowledge and understanding about the biological functions of miR393. The data obtained in a perennial crop species provide critical information for the development of new biotechnology approaches for genetically engineering other important crops for enhanced agricultural production.

## Experimental procedures

### Cloning of the *Osa‐miR393a* gene and construction of overexpression vector

To make the *Osa‐miR393a* overexpression construct, the full‐length rice (*Oryza sativa*) *Osa‐miR393a* cDNA fragment containing a stem‐loop structure was amplified by PCR using the forward and reverse primer set and cloned into the binary vector pZH01 (Xiao *et al*., 2003), under the CaMV35S promoter and the CaMV35S promoter driving the *hyg* gene for hygromycin resistance as a selectable marker. The construct containing the overexpression cassette for *Osa‐miR393a* was transformed into the *Agrobacterium tumefaciens* strain LBA4404 by electroporation. Table S1 has summarized all the primers used in this study.

### Plant materials and transformation

The transformed *Agrobacterium* containing the overexpression construct of *Osa‐miR393a* was used to infect the embryonic callus induced from the mature seeds of creeping bentgrass (*A. stolonifera* L.) cv. Penn A‐4 (Supplied by HybriGene, Hubbard, OR, USA) following the previous protocol (Luo *et al*., 2004).

### Plant propagation, maintenance and abiotic stress treatments

The newly generated transgenic creeping bentgrass plants overexpressing *Osa‐miR393a* and wild‐type controls were subject to clonal propagation by multiplying their tillers. All the plants were maintained as described previously (Yuan *et al*., 2015; Zhou *et al*., 2013).

To investigate the plant response to salt stress, plants grown in cone‐tainers and small‐sized pots were immersed in NaCl solution supplemented with 0.2 g/L water‐soluble fertilizer (20 : 10 : 20 nitrogen : phosphorus : potassium; Peat‐Lite Special; Scotts). Based on literatures and our previous work, a concentration of 200 mm NaCl is adequate for salt stress test in creeping bentgrass. Considering that plant materials for salt test were grown in pure silicon sand, which contains water, we decided to use an increased NaCl concentration of 250 mm for plant salt treatment. Shoots were harvested 10 days after salt treatments for further physiological analysis. Replicates of the salt‐treated plants were watered with 0.2 g/L water‐soluble fertilizer every other day for recovery from salt stress and documented by photography. Wild‐type leaves were collected at 0, 1.5, 3 and 6 h after salt stress treatment for analysis of miR393 expression.

To evaluate heat stress tolerance, plants grown in cone‐tainers and Elite 1200 pots, respectively, were immersed in 0.2 g/L water‐soluble fertilizer in a growth chamber (Conviron, Controlled Environments Inc., Pembina, ND) and subjected to heat stress by heating the plants to 40 °C in the light and 35 °C in the dark for 14 days. The relative humidity in the chamber was 60%–80%, and the heat‐stressed plants were well‐watered every 2 days with distilled water. wild‐type leaves were collected at 0, 1.5, 3 and 6 h after heat stress treatment for analysis of miR393 expression.

To study plant response to drought stress, two events of TG (TG1 and TG4) and WT control plants were vegetatively propagated from stolons or a single tiller of the same size (limited water supply) as previously described (Li *et al*., 2013; Zhou *et al*., 2013). The plants in the big‐sized pots (10 cm × 10.5 cm; Dillen Products, Middlefield, OH) or cone‐tainers with pure sand were maintained under normal conditions in growth room for 10 weeks and then subjected to drought stress by water withholding or limited water supply (10 mL for each plant every 2 days). For miR393 expression analysis, wild‐type plants taken out of the pure sand were dried with a paper towel and then laid on the bench for air drying. The leaf samples were collected at 0, 1.5, 3 and 6 h after drought treatment.

Wild‐type leaves were collected at 0, 1.5, 3 and 6 h after 50 μm indole‐3‐acetic acid (IAA) solution treatment for analysis of miR393 expression.

### Identification of transgenic plants, isolation of plant RNA and gene expression analysis

Genomic DNA was isolated from the putative transgenic and wild‐type plants. The PCR was used to screen for putative transgenic plants with the primers *Hyg*‐F and *Hyg*‐R (Table S1).

Total RNA was extracted from plant leaf with the Trizol reagent and treated with RNase‐free DNaseI (Invitrogen, Carlsbad, CA). First‐strand cDNA was synthesized using SuperScript III Reverse Transcriptase Kit (Invitrogen) following the manufacturer's protocol. *AsUBQ* was used as the internal reference for assessing gene expression level in creeping bentgrass. Semi‐quantitative RT–PCR was conducted using the following program: 5‐min denaturation at 94 °C, followed by 24 to 30 cycles of 94 °C for 30 s, 60 °C for 30 s and 72 °C for 30 s and completed with an extension step of 5 min at 72 °C. Real‐time RT–PCR was conducted in a total volume of 25 μL containing 12.5 μL of iQ SYBR‐Green Supermix (Bio‐Rad Laboratories) on the Bio‐RadiQ5 real‐time detection system according to the manufacturer's instructions using the following program: 5 min denaturation at 94 °C, followed by 40 cycles of 94 °C for 30 s, 60 °C for 30 s and 72 °C for 30 s. The measurements were obtained using the relative quantification method (Livak and Schmittgen, 2012). The primers used were listed in Table S1. Stem‐loop RT–qPCR was performed according to the protocol of Varkonyi‐Gasic *et al*. (2007).

### Measurement of mineral content, leaf RWC, EL, chlorophyll and MDA content

To examine Na^+^ and K^+^ contents, plants were grown hydroponically to facilitate root sampling. Under this condition, plant roots were in direct contact with NaCl solution, we therefore used 200 mm, a lower concentration of NaCl than that for plants grown in sands, for salt treatment. The leaves and roots of the wild‐type and transgenic plants were collected before and after a 10‐day treatment of 200 mm NaCl. A total of 0.5 g dried materials was used in each sample. The whole procedure of determination of the mineral contents in plants was described in previous protocols (Haynes, 1980; Li *et al*., 2010).

Plant leaf RWC, EL, chlorophyll content and MDA content were measured following previous protocols (Bates *et al*., 1973; Dhindsa *et al*., 1981; Li *et al*., 2010).

### Measurement of water loss rate

To detect the water loss rate under dehydration conditions, transgenic plants and wild‐type plants were exposed to air at room temperature and weighed at the designated times.

### Plant histological analysis

Histological analysis for the leaf and stem cross sections was performed as described previously (Yuan *et al*., 2015). In this study, sections were stained using Toluidine blue. For leaf cuticle features observation, leaves of the same age at the same relative positions sampled from the longest tiller of the wild‐type and transgenic plants were fixed in 2.5% (vol/vol) glutaraldehyde. Samples were sputter‐coated with gold particles and coated surfaces were viewed using a JEOL JSM‐6390LV scanning electron microscope.

### Measurements of leaf stomatal density

For stomatal density measurement, leaves of the same age at the same relative positions were sampled from the longest tiller of the wild‐type and transgenic plants. A drop of clear nail polish was applied to the leaf upper epidermis and let it dry completely. The films were then peeled off and observed under a light microscope (MEIJI EMZ‐5TR).

### cDNA library preparation and Illumina sequencing, differential expression and GO enrichment analyses

Total RNA was isolated from leaves of wild‐type and *Osa‐miR393a* TG4 plants grown under normal conditions with Trizol reagent (Invitrogen). Poly (A)‐containing mRNA molecules were purified from total RNA using poly (T) oligo‐attached magnetic beads and then fragmented into 150‐ to 250‐bp pieces using fragmentation reagent, and the first‐strand cDNA was generated using random hexamer‐primed reverse transcription. The second‐strand cDNA was generated upon completion of the first‐strand synthesis with dTTP being replaced by dUTP. The synthesized cDNA was subsequently subjected to end‐repair followed by 3′ adenylation. Adaptors were ligated to the ends of these 3′‐adenylated cDNA fragments, and the second‐strand cDNA degradation was performed by UDG (uracil–DNA glycocasylase). The products of the ligation reaction were purified on TAE‐agarose gel. Many rounds of PCR amplification were performed to enrich the purified cDNA template. The cDNA library was validated by the Agilent Technologies 2100 Bio‐analyser and the ABI Step One Plus Real‐Time PCR System. Paired‐end sequencing of each library was performed using the HiSeq 2000 (Illumina Technologies) platform following the manufacturer's instructions. The removal of poor quality sequences and trimming of adaptor sequences from the raw sequence data were carried out using cut adapt (v1.8.1). The clean sequencing data for each sample were assembled to transcripts using Trinity software (Haas *et al*., 2013). All assembled transcripts from total samples were clustered by cdhit (v4.6) with default parameters (Li and Godzik, 2006). The clustered transcripts were then aligned to Rfam database (v.11) (http://www.sanger.ac.uk/Software/Rfm/) to exclude those matching rRNAs. The open‐reading frame regions of transcripts were predicted by the Transdecoder (v.2.0.1). RSEM method was adopted to quantify total expressed transcripts with the TPM (Transcripts per million) value (Li and Dewey, 2011). Tool edgeR package was used to identify the differential expression genes on the read count values of genes (Nikolayeva and Robinson, 2014). The fold change between the two groups was calculated as: logFC = log2 (transgenic group/wild group). Gene in two groups, whose |logFC| > 1 and *q* value <0.05 (it depends), was defined as differential expression genes in this study. The ‘blastp’ program (v.2.2.29) was used to annotate these expressed transcripts’ function with NR, NOG and swissport database with the cut‐off of *e*‐value <1e−5. The software of InterProScan (v.5.15‐54.0) was used to annotate the domains, gene families and Gene Ontology (GO) functions. GO classification was used to obtain GO functional class for all the transcripts.

## Conflict of interest

The authors declare no conflict of interests.

## Supporting information


**Figure S1** Expression profiles analysis of miR393 by stem‐loop RT–qPCR in response to salt, drought, heat and IAA in wild‐type creeping bentgrass. Relative expression levels of the mature miR393 were determined in different tissues (a), and in leaves under salt (b), drought (c), heat (d), and IAA (e) treatment. Data are presented as means of three biological replicates, and error bars represent SD.
**Figure S2** Generation and molecular analysis of transgenic lines expressing *Osa‐miR393a*. (a) Schematic diagram of the *Osa‐miR393a* overexpression construct, p35S‐*Osa‐miR393a*/p35S‐*Hyg*. *Osa‐miR393a* is under the control of the *Cauliflower mosaic virus* (CaMV) 35S promoter and linked to the hygromycin resistance gene, *Hyg*, driven by the CaMV 35S promoter. LB, Left border; RB, right border; NOS, nopaline synthase terminator. (b) PCR analysis of the *Hyg* gene using genomic DNA of wild‐type and transgenic plants to detect transgene insertion into the host genome. (c) Semi‐quantitative RT–PCR analysis of the primary *Osa‐miR393a* transcripts in transgenics. (d) Stem‐loop RT–qPCR analysis to detect the expression of mature *miR393* in transgenic and wild‐type plants. Data are presented as means of three biological replicates, and error bars represent SD. Asterisks indicate a significant difference of expression levels between the wild‐type and each transgenic line at *P <* *0.001 by Student's *t*‐test.
**Figure S3** The phenotype of two‐month‐old wild‐type and transgenic plants initiated from the same number of tillers and grown in 6‐inch pots.
**Figure S4** Overexpression of Osa‐m*iR393a* leads to enhanced salt tolerance in transgenic turfgrass plants. (a) Wild‐type controls and three transgenic lines initiated from the same number of tillers were fully developed in pots for 10 weeks under normal conditions in growth room before salt stress application. (b) Performance of wild‐type and transgenic plants subjected to 250 mm NaCl treatment for 10 days. (c) Performance of wild‐type and transgenic plants 7 days after recovery from a 10 days salt treatment.
**Figure S5** Overexpression of *Osa‐miR393a* leads to enhanced heat tolerance in transgenic turfgrass plants. Wild‐type controls and three transgenic lines were fully developed in pots for 10 weeks under normal conditions in growth room. Plants were then transferred to the growth chamber and subjected to heat stress at 40 °C in the light and 35 °C in the dark for 15 days. The relative humidity in the chamber was 60%–80%. (a) and (c) Wild‐type controls and three transgenic lines initiated from the same number of tillers were trimmed to the same height before the heat stress test. (b) Performance of wild‐type and transgenic plants grown at 25 °C in the light and 17 °C in the dark for 15 days. (d) Performance of wild‐type and transgenic plants 10 days after recovery from a 15 days heat treatment at 40 °C in the light and 35 °C in the dark.
**Figure S6** Shoot and root biomass change in wild‐type (WT) and transgenic (TG) plants subjected to heat stress. (a) Performance of wild‐type plants and three independent transgenic lines 13 days after growth under 25 °C/17 °C or 40 °C/35 °C. (b) Shoot fresh weight and (c) dry weight in wild‐type and three independent transgenic lines 13 days after growth under 25 °C/17 °C or 40 °C/35 °C. (d) Root fresh weight and (e) dry weight in wild‐type and three independent transgenic lines 13 days after growth under 25 °C/17 °C and 40 °C/35 °C. Data are presented as means of three biological replicates, and error bars represent SD.
**Figure S7** Amino acid sequences alignment of *AsTIR1*,* AtTIR1* and *OsTIR1*.
**Figure S8** Amino acid sequences alignment of *AsAFB2‐1*,* AsAFB2‐2*,* AtAFB2* and *OsAFB2*.
**Figure S9** Differential gene expression in transgenic (TG) vs. wild‐type (WT) control plants. Volcano plot shows log_2_ FC of TG vs. WT data sets at normal conditions.
**Figure S10** GO enrichment analysis in transgenic (TG) vs. wild‐type (WT) control plants.
**Figure S11** Expression levels of (a) *AsNHX1* and (b) *AsVP1* in wild‐type (WT) and transgenic (TG) plants examined via qRT–PCR. Their differential expression between wild‐type (WT) and transgenic (TG) plants was initially revealed by RNA‐seq data. Data are presented as means of three biological replicates, and error bars represent SD. Asterisks indicate a significant difference of expression levels between the wild‐type and transgenic plants at *P <* *0.001 by Student's *t*‐test.
**Figure S12** Relative expression levels of the two putative miR393 targets of creeping bentgrass were determined in leaves under IAA treatment. Data are presented as means of three biological replicates, and error bars represent SD. Asterisks indicate significant differences of gene expression levels between untreated and stress‐treated leaf tissues: *P <* *0.05; and ***P <* *0.001 by Student's *t*‐test.

**Table S1** Primer sequences used in this study.
**Table S2** The sequence of putative target genes for *miR393* in creeping bentgrass, target sites are shown in red.
