# Supplementary material for: Transgenic creeping bentgrass overexpressing Osa‐miR393a exhibits altered plant development and improved multiple stress tolerance
Source: Plant Biotechnol J. 2018 Jul 4;17(1):233–51. doi: 10.1111/pbi.12960 (PMC6330543; doi:10.1111/pbi.12960)
Supplement: Supplementary file 1 — Figure S1 Expression profiles analysis of miR393 by stem‐loop RT–qPCR in response to salt, drought, heat and IAA in wild‐type creeping bentgrass. Relative expression levels of the mature miR393 were determined in different tissues (a), and in leaves under salt (b), drought (c), heat (d), and IAA (e) treatment. Data are presented as means of three biological replicates, and error bars represent SD. Figure S2 Generation and molecular analysis of transgenic lines expressing Osa‐miR393a. (a) Schematic diagram of the Osa‐miR393a overexpression construct, p35S‐Osa‐miR393a/p35S‐Hyg. Osa‐miR393a is under the control of the Cauliflower mosaic virus (CaMV) 35S promoter and linked to the hygromycin resistance gene, Hyg, driven by the CaMV 35S promoter. LB, Left border; RB, right border; NOS, nopaline synthase terminator. (b) PCR analysis of the Hyg gene using genomic DNA of wild‐type and transgenic plants to detect transgene insertion into the host genome. (c) Semi‐quantitative RT–PCR analysis of the primary Osa‐miR393a transcripts in transgenics. (d) Stem‐loop RT–qPCR analysis to detect the expression of mature miR393 in transgenic and wild‐type plants. Data are presented as means of three biological replicates, and error bars represent SD. Asterisks indicate a significant difference of expression levels between the wild‐type and each transgenic line at *P < 0.001 by Student's t‐test. Figure S3 The phenotype of two‐month‐old wild‐type and transgenic plants initiated from the same number of tillers and grown in 6‐inch pots. Figure S4 Overexpression of Osa‐miR393a leads to enhanced salt tolerance in transgenic turfgrass plants. (a) Wild‐type controls and three transgenic lines initiated from the same number of tillers were fully developed in pots for 10 weeks under normal conditions in growth room before salt stress application. (b) Performance of wild‐type and transgenic plants subjected to 250 mm NaCl treatment for 10 days. (c) Performance of wild‐type and transgenic plants 7 [file PBI-17-233-s002.pptx]

## Slide 1
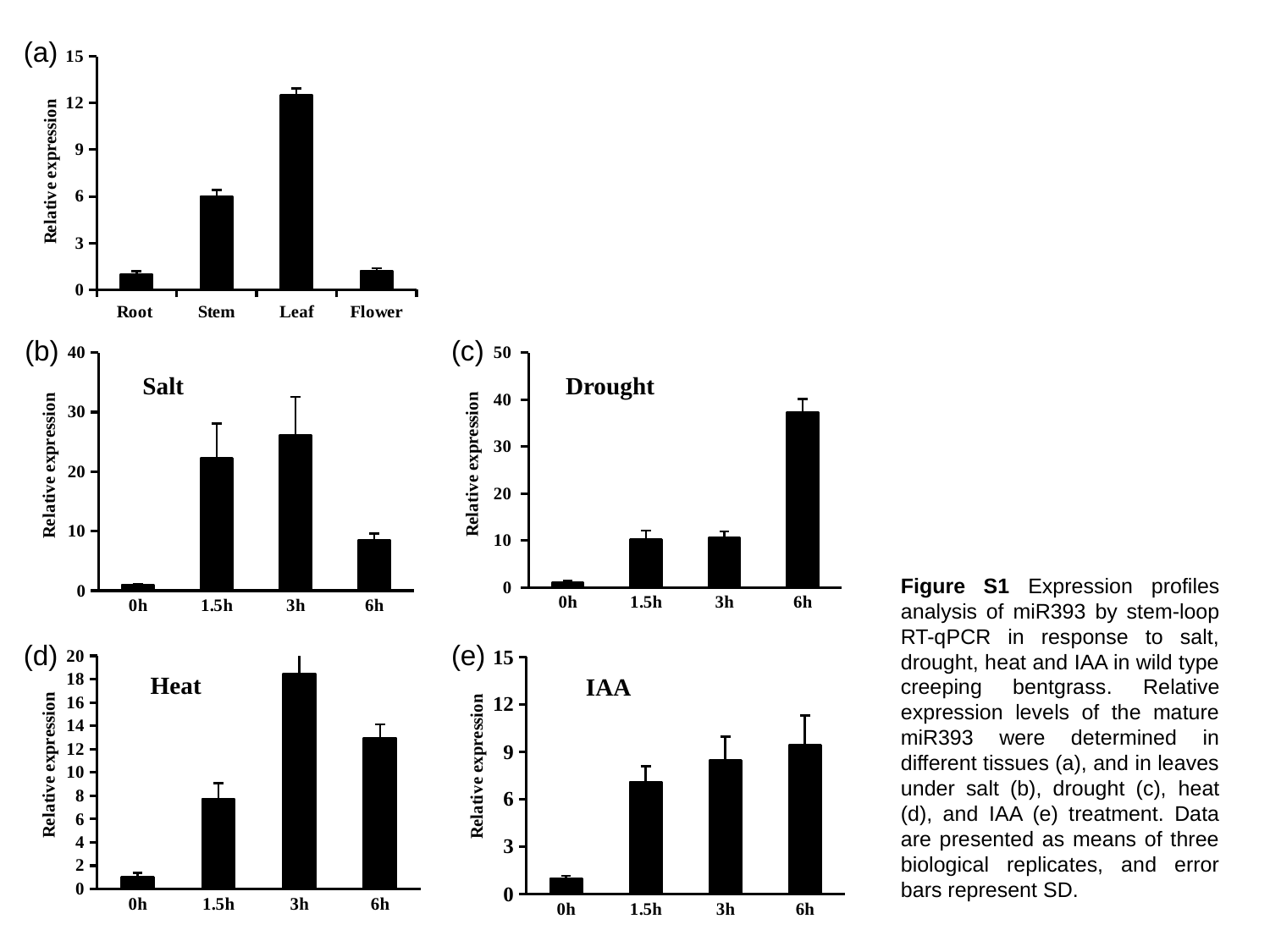

(a)
### Chart
| Category | |
|---|---|
| Root | 1.005655067393401 |
| Stem | 5.999252436125336 |
| Leaf | 12.50249822153692 |
| Flower | 1.213790148959655 |
### Chart
| Category | |
|---|---|
| 0h | 1.007902051202455 |
| 1.5h | 22.27838360896744 |
| 3h | 26.17007145397396 |
| 6h | 8.52203268592795 |Salt
### Chart
| Category | |
|---|---|
| 0h | 1.023477075104296 |
| 1.5h | 10.21603877195893 |
| 3h | 10.58341408051019 |
| 6h | 37.30243208266329 |Drought
### Chart
| Category | |
|---|---|
| 0h | 1.023477075104296 |
| 1.5h | 7.73569049529243 |
| 3h | 18.46284809670746 |
| 6h | 12.9395240325754 |Heat
### Chart
| Category | |
|---|---|
| 0h | 1.007902051202455 |
| 1.5h | 7.10415700745153 |
| 3h | 8.46768139672565 |
| 6h | 9.439738122441296 |IAA
(b)
(c)
(d)
(e)
Figure S1 Expression profiles analysis of miR393 by stem-loop RT-qPCR in response to salt, drought, heat and IAA in wild type creeping bentgrass. Relative expression levels of the mature miR393 were determined in different tissues (a), and in leaves under salt (b), drought (c), heat (d), and IAA (e) treatment. Data are presented as means of three biological replicates, and error bars represent SD.

## Slide 2
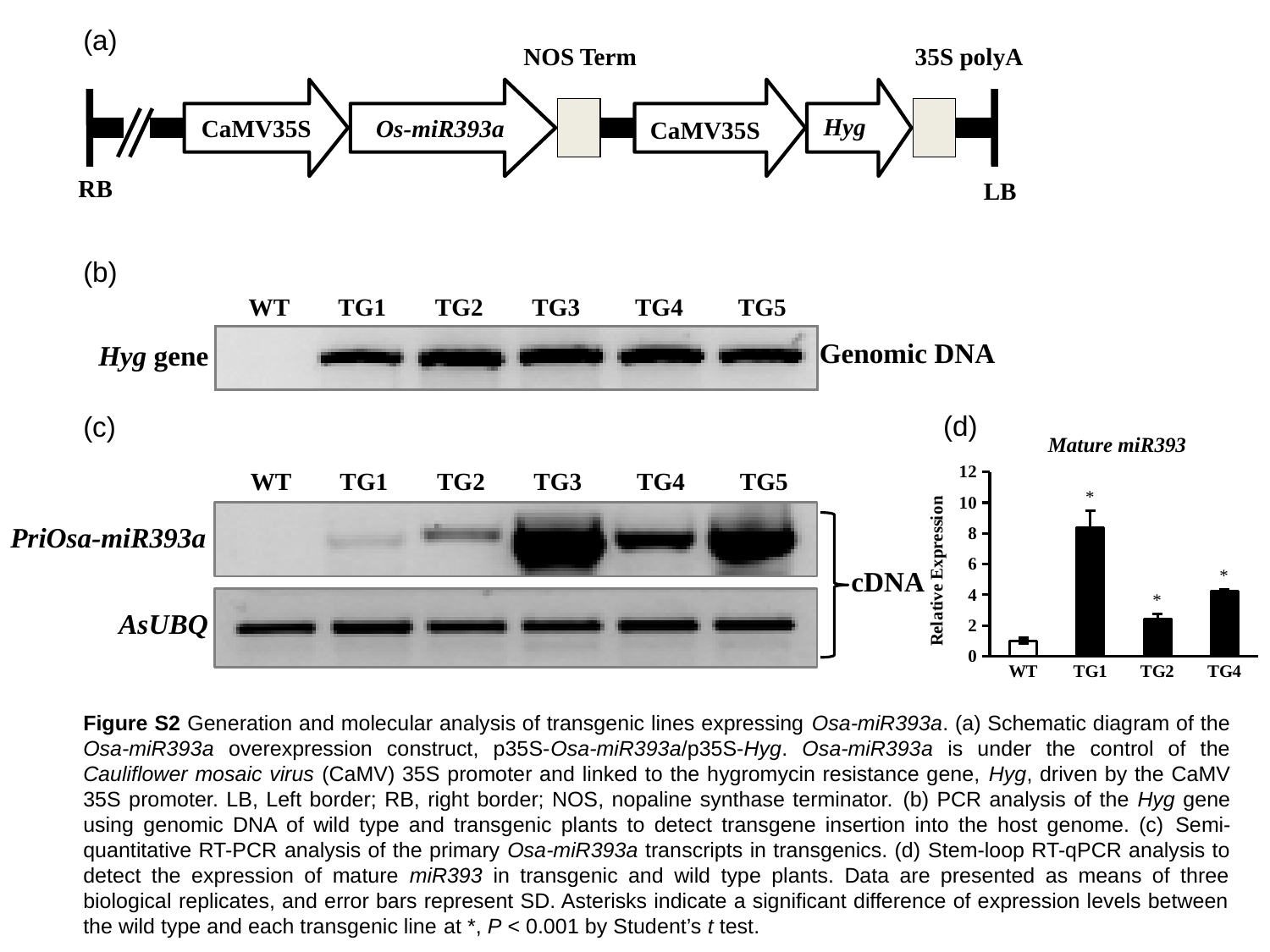

(a)
35S polyA
NOS Term
CaMV35S
Os-miR393a
Hyg
CaMV35S
RB
LB
(b)
 WT TG1 TG2 TG3 TG4 TG5
Hyg gene
Mature miR393
 WT TG1 TG2 TG3 TG4 TG5
PriOsa-miR393a
AsUBQ
Genomic DNA
(d)
(c)
### Chart
| Category | |
|---|---|
| WT | 1.005421690468437 |
| TG1 | 8.37751146156891 |
| TG2 | 2.433335804481151 |
| TG4 | 4.226818966485645 |
cDNA
*
*
*
Figure S2 Generation and molecular analysis of transgenic lines expressing Osa-miR393a. (a) Schematic diagram of the Osa-miR393a overexpression construct, p35S-Osa-miR393a/p35S-Hyg. Osa-miR393a is under the control of the Cauliflower mosaic virus (CaMV) 35S promoter and linked to the hygromycin resistance gene, Hyg, driven by the CaMV 35S promoter. LB, Left border; RB, right border; NOS, nopaline synthase terminator. (b) PCR analysis of the Hyg gene using genomic DNA of wild type and transgenic plants to detect transgene insertion into the host genome. (c) Semi-quantitative RT-PCR analysis of the primary Osa-miR393a transcripts in transgenics. (d) Stem-loop RT-qPCR analysis to detect the expression of mature miR393 in transgenic and wild type plants. Data are presented as means of three biological replicates, and error bars represent SD. Asterisks indicate a significant difference of expression levels between the wild type and each transgenic line at *, P < 0.001 by Student’s t test.

## Slide 3
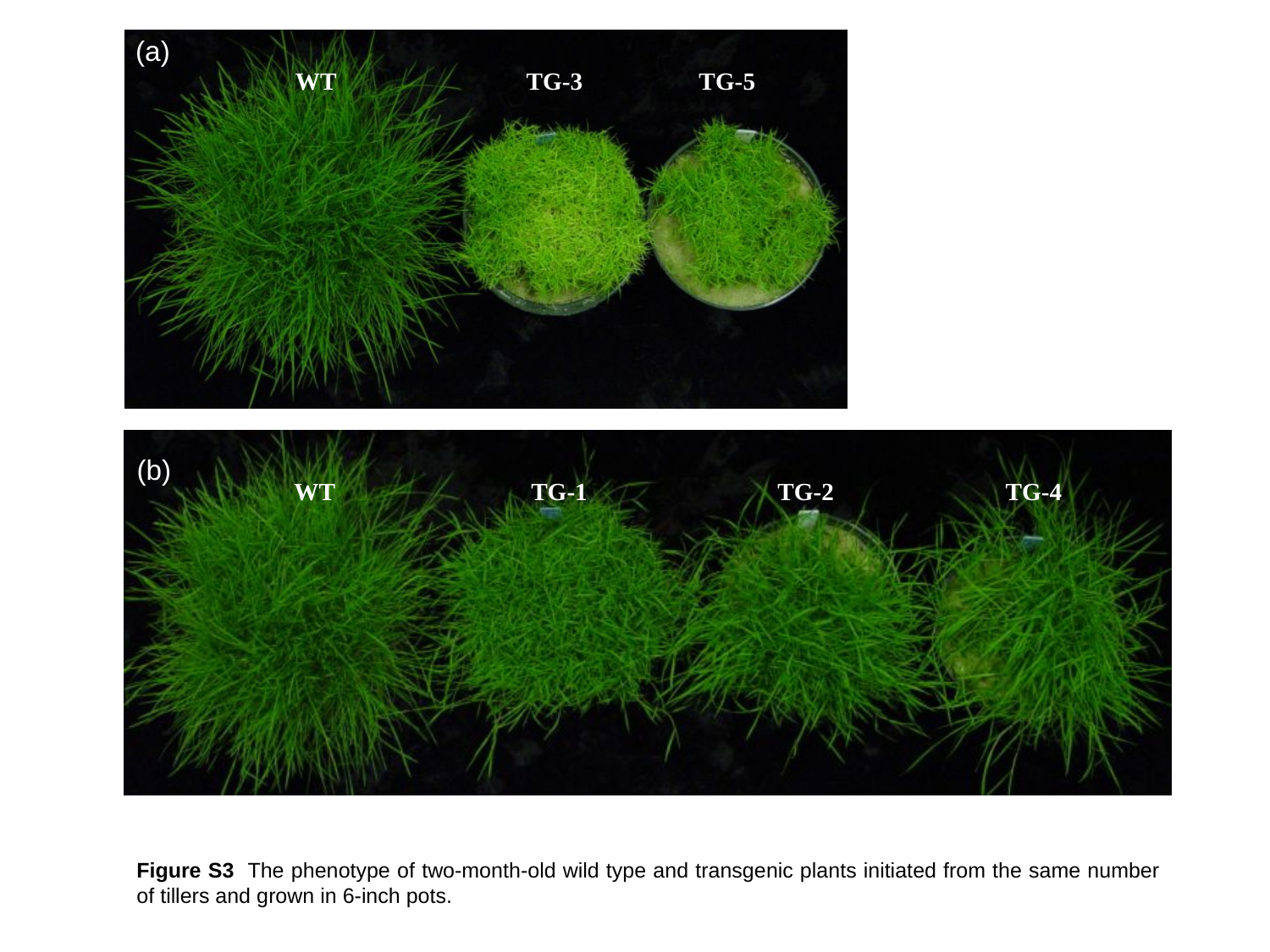

(a)
WT TG-3 TG-5
(b)
WT TG-1 TG-2 TG-4
Figure S3 The phenotype of two-month-old wild type and transgenic plants initiated from the same number of tillers and grown in 6-inch pots.

## Slide 4
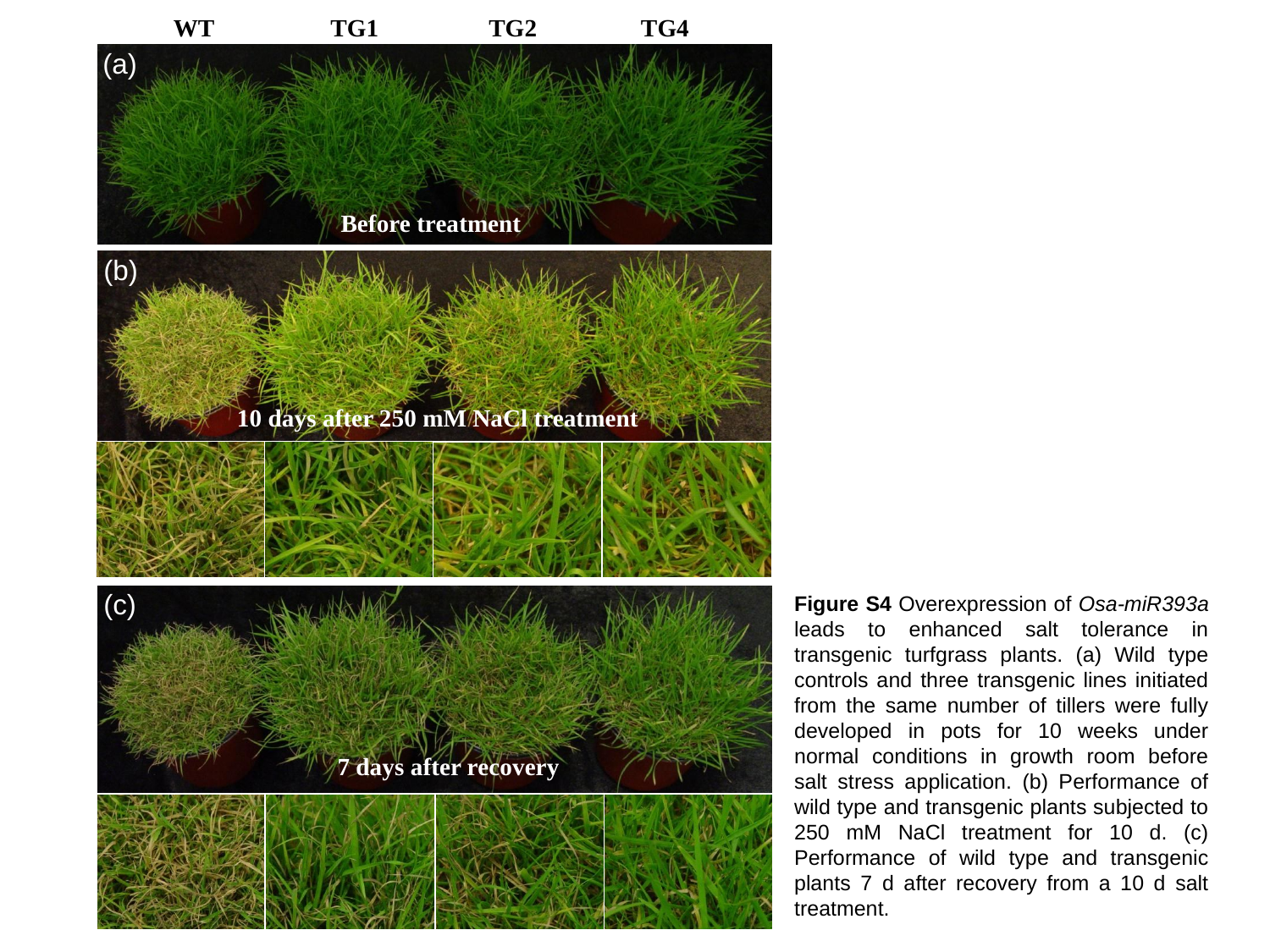

WT TG1 TG2 TG4
Before treatment
10 days after 250 mM NaCl treatment
7 days after recovery
(a)
(b)
(c)
Figure S4 Overexpression of Osa-miR393a leads to enhanced salt tolerance in transgenic turfgrass plants. (a) Wild type controls and three transgenic lines initiated from the same number of tillers were fully developed in pots for 10 weeks under normal conditions in growth room before salt stress application. (b) Performance of wild type and transgenic plants subjected to 250 mM NaCl treatment for 10 d. (c) Performance of wild type and transgenic plants 7 d after recovery from a 10 d salt treatment.

## Slide 5
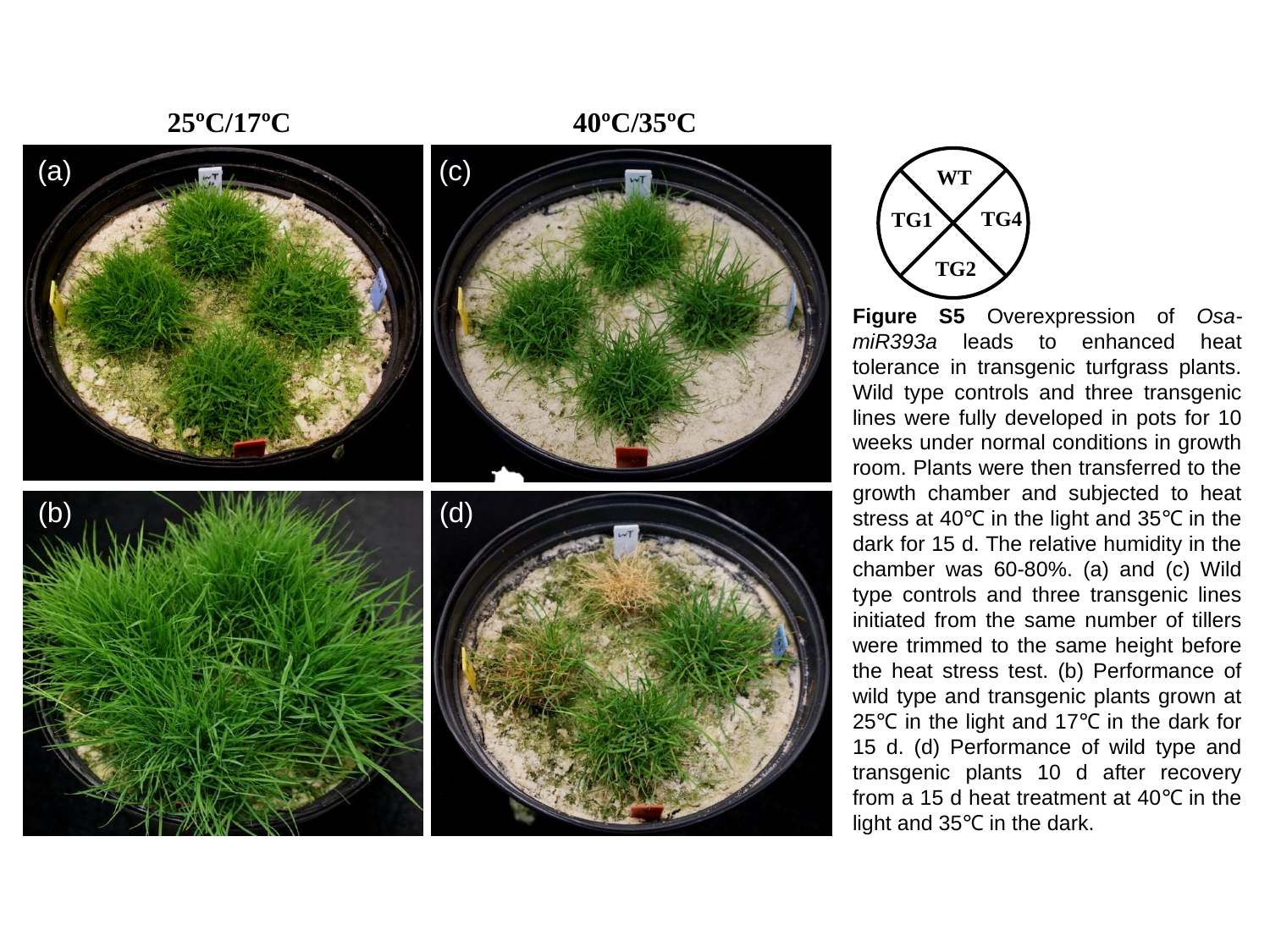

25ºC/17ºC
40ºC/35ºC
WT
TG4
TG1
TG2
(a) (c)
(b) (d)
Figure S5 Overexpression of Osa-miR393a leads to enhanced heat tolerance in transgenic turfgrass plants. Wild type controls and three transgenic lines were fully developed in pots for 10 weeks under normal conditions in growth room. Plants were then transferred to the growth chamber and subjected to heat stress at 40℃ in the light and 35℃ in the dark for 15 d. The relative humidity in the chamber was 60-80%. (a) and (c) Wild type controls and three transgenic lines initiated from the same number of tillers were trimmed to the same height before the heat stress test. (b) Performance of wild type and transgenic plants grown at 25℃ in the light and 17℃ in the dark for 15 d. (d) Performance of wild type and transgenic plants 10 d after recovery from a 15 d heat treatment at 40℃ in the light and 35℃ in the dark.

## Slide 6
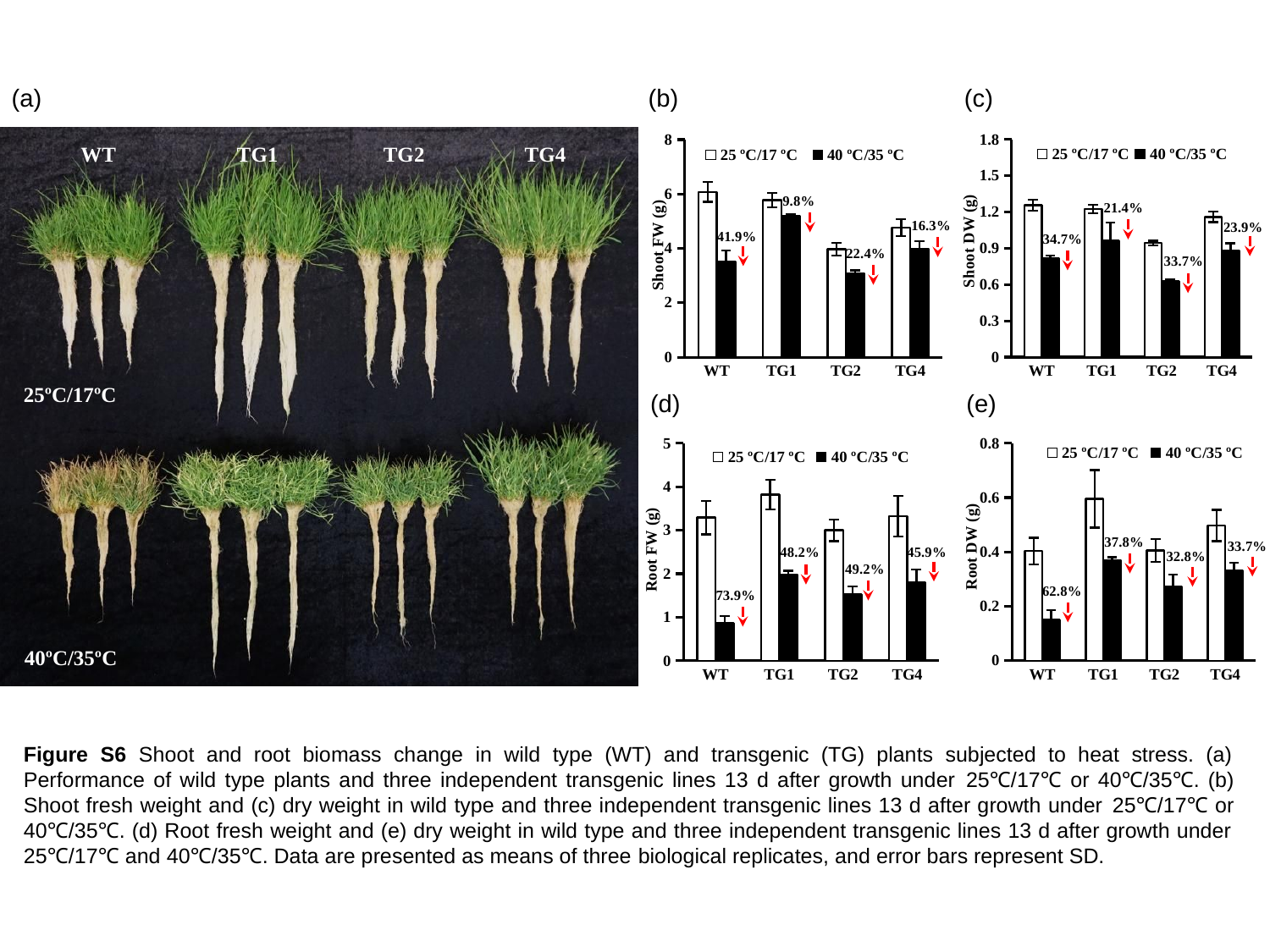

(a)
 (b) (c)
 WT TG1 TG2 TG4
25ºC/17ºC
40ºC/35ºC
### Chart
| Category | 25 ºC/17 ºC | 40 ºC/35 ºC |
|---|---|---|
| WT | 1.256666666666667 | 0.82 |
| TG1 | 1.225 | 0.963333333333333 |
| TG2 | 0.943333333333333 | 0.625 |
| TG4 | 1.16 | 0.883333333333333 |21.4%
23.9%
34.7%
33.7%
### Chart
| Category | 25 ºC/17 ºC | 40 ºC/35 ºC |
|---|---|---|
| WT | 6.073333333333335 | 3.526666666666667 |
| TG1 | 5.77 | 5.205 |
| TG2 | 3.965 | 3.075 |
| TG4 | 4.756666666666667 | 3.98 |9.8%
16.3%
41.9%
22.4%
### Chart
| Category | 25 ºC/17 ºC | 40 ºC/35 ºC |
|---|---|---|
| WT | 0.403333333333333 | 0.15 |
| TG1 | 0.595 | 0.37 |
| TG2 | 0.405 | 0.272 |
| TG4 | 0.4975 | 0.33 |37.8%
33.7%
32.8%
62.8%
### Chart
| Category | 25 ºC/17 ºC | 40 ºC/35 ºC |
|---|---|---|
| WT | 3.29 | 0.86 |
| TG1 | 3.82 | 1.98 |
| TG2 | 3.0 | 1.524 |
| TG4 | 3.3275 | 1.8 |48.2%
45.9%
49.2%
73.9%
 (d) (e)
Figure S6 Shoot and root biomass change in wild type (WT) and transgenic (TG) plants subjected to heat stress. (a) Performance of wild type plants and three independent transgenic lines 13 d after growth under 25℃/17℃ or 40℃/35℃. (b) Shoot fresh weight and (c) dry weight in wild type and three independent transgenic lines 13 d after growth under 25℃/17℃ or 40℃/35℃. (d) Root fresh weight and (e) dry weight in wild type and three independent transgenic lines 13 d after growth under 25℃/17℃ and 40℃/35℃. Data are presented as means of three biological replicates, and error bars represent SD.

## Slide 7
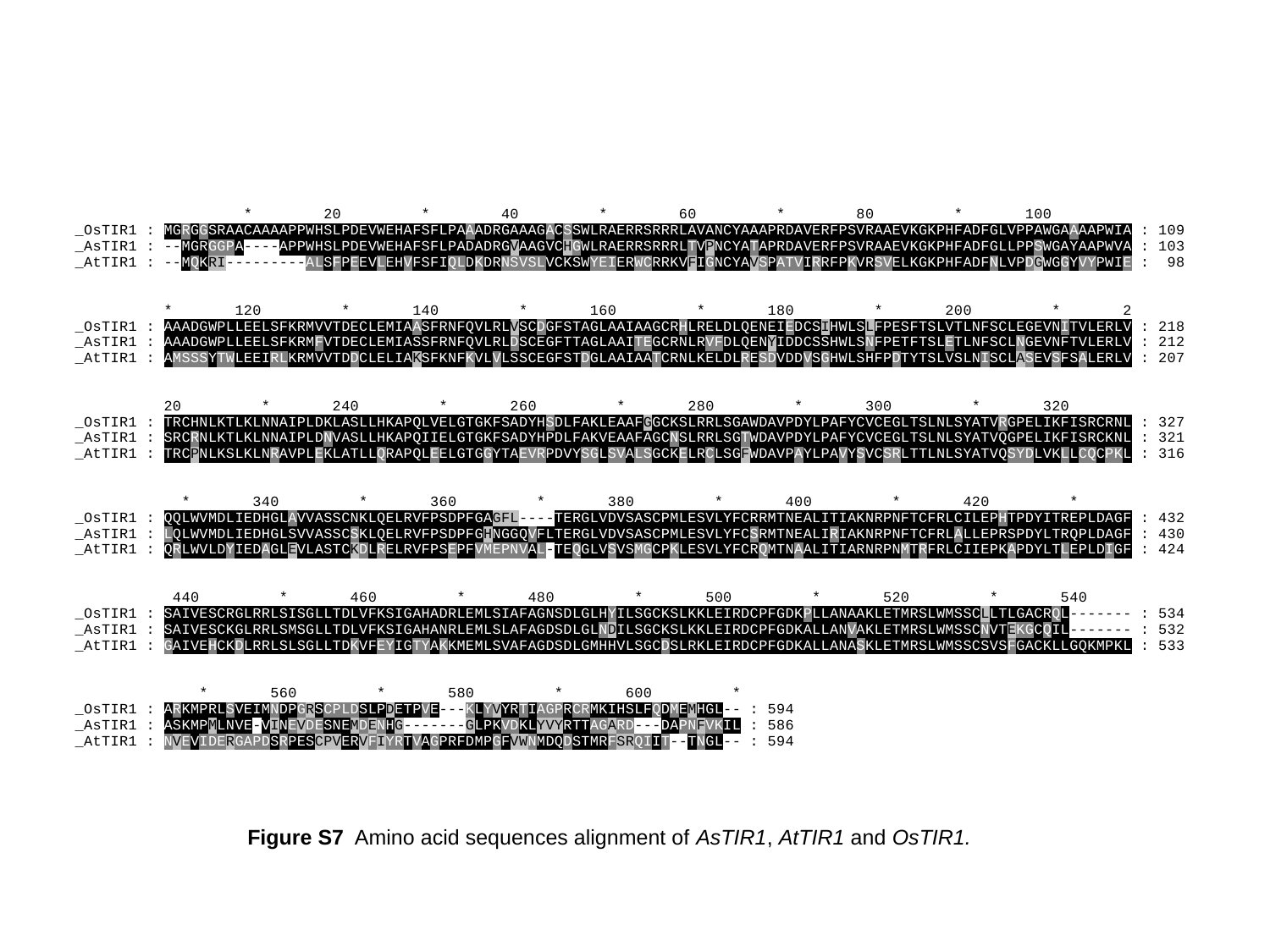

Figure S7 Amino acid sequences alignment of AsTIR1, AtTIR1 and OsTIR1.

## Slide 8
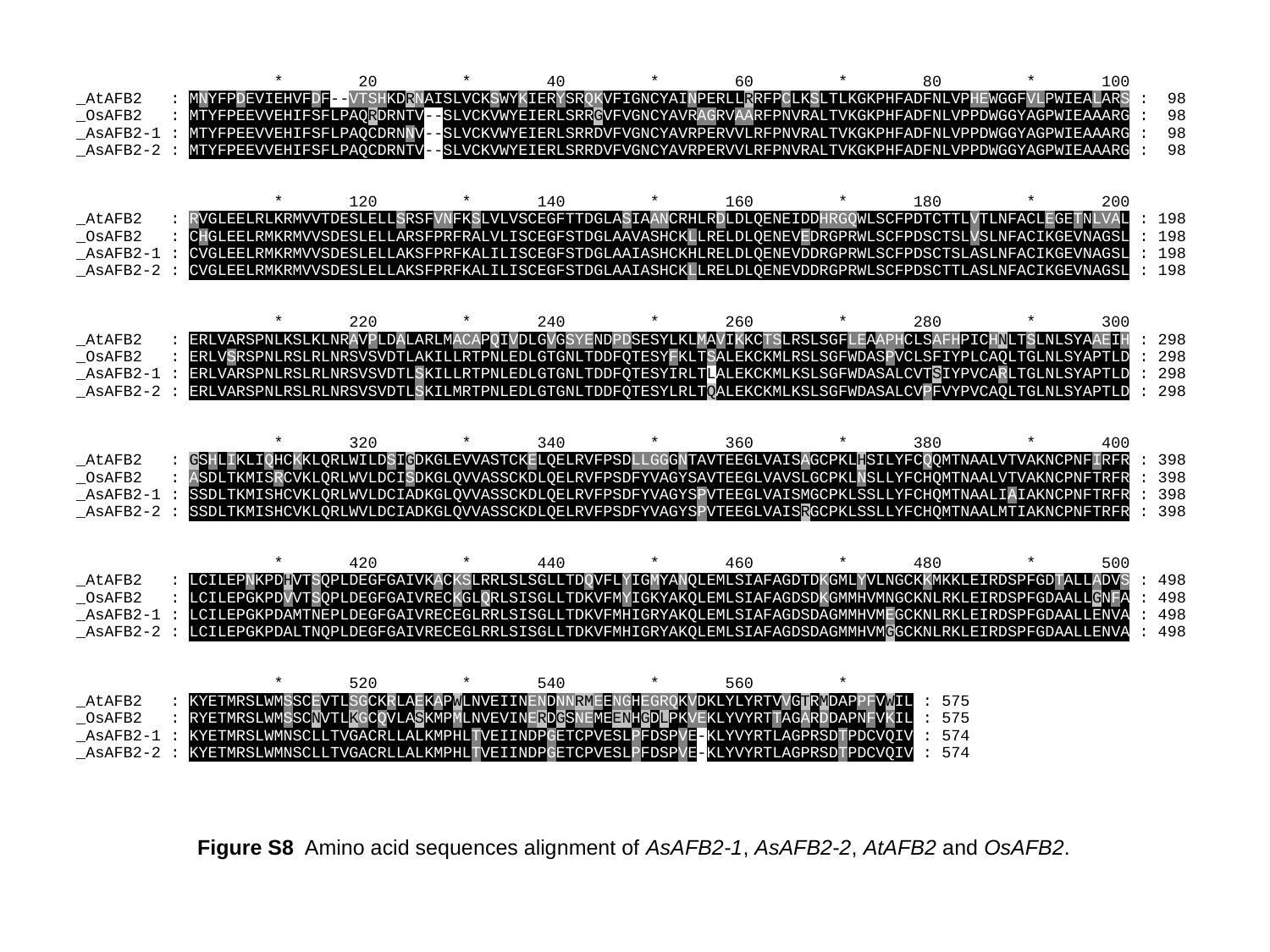

Figure S8 Amino acid sequences alignment of AsAFB2-1, AsAFB2-2, AtAFB2 and OsAFB2.

## Slide 9
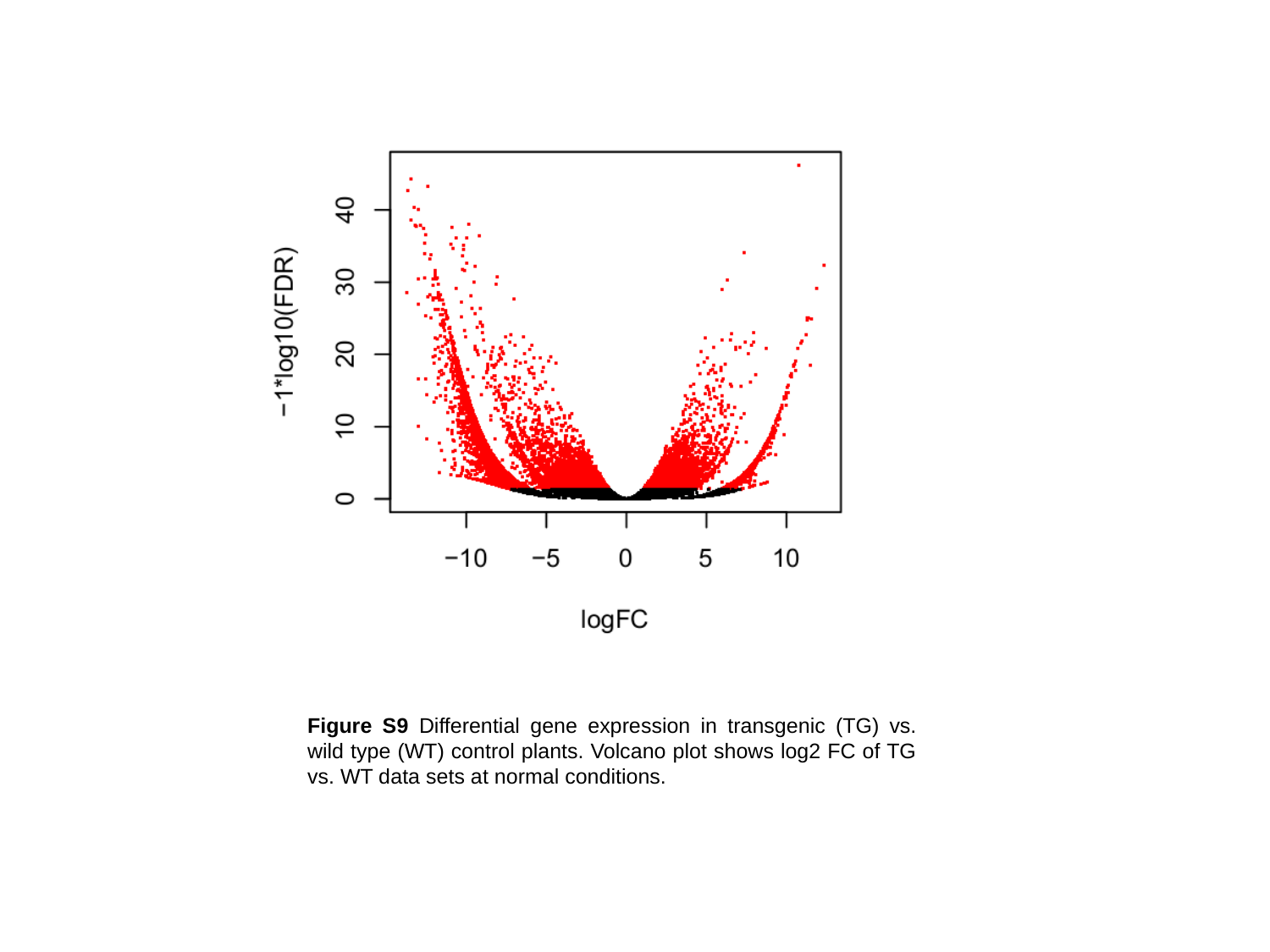

Figure S9 Differential gene expression in transgenic (TG) vs. wild type (WT) control plants. Volcano plot shows log2 FC of TG vs. WT data sets at normal conditions.

## Slide 10
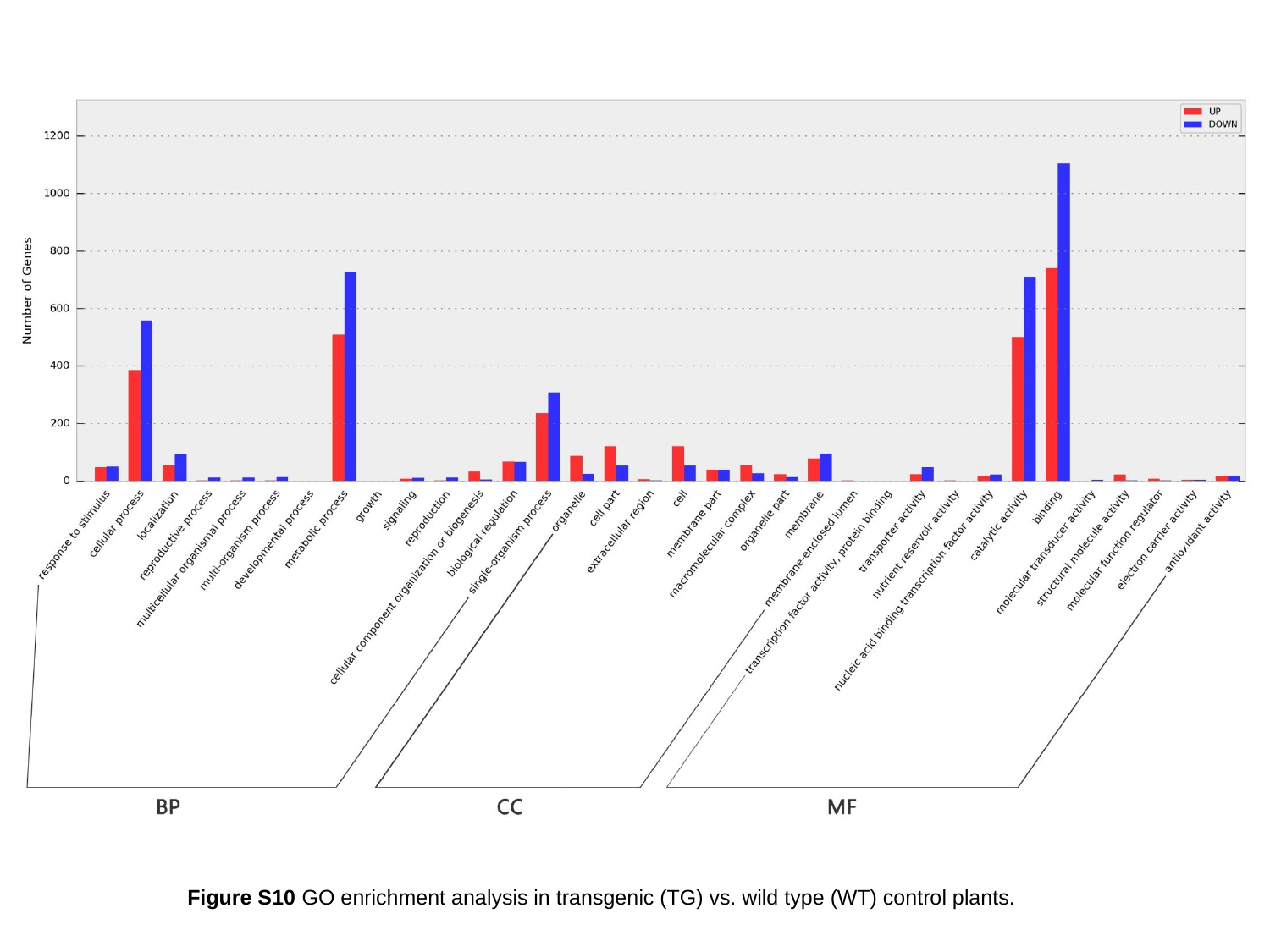

Figure S10 GO enrichment analysis in transgenic (TG) vs. wild type (WT) control plants.

## Slide 11
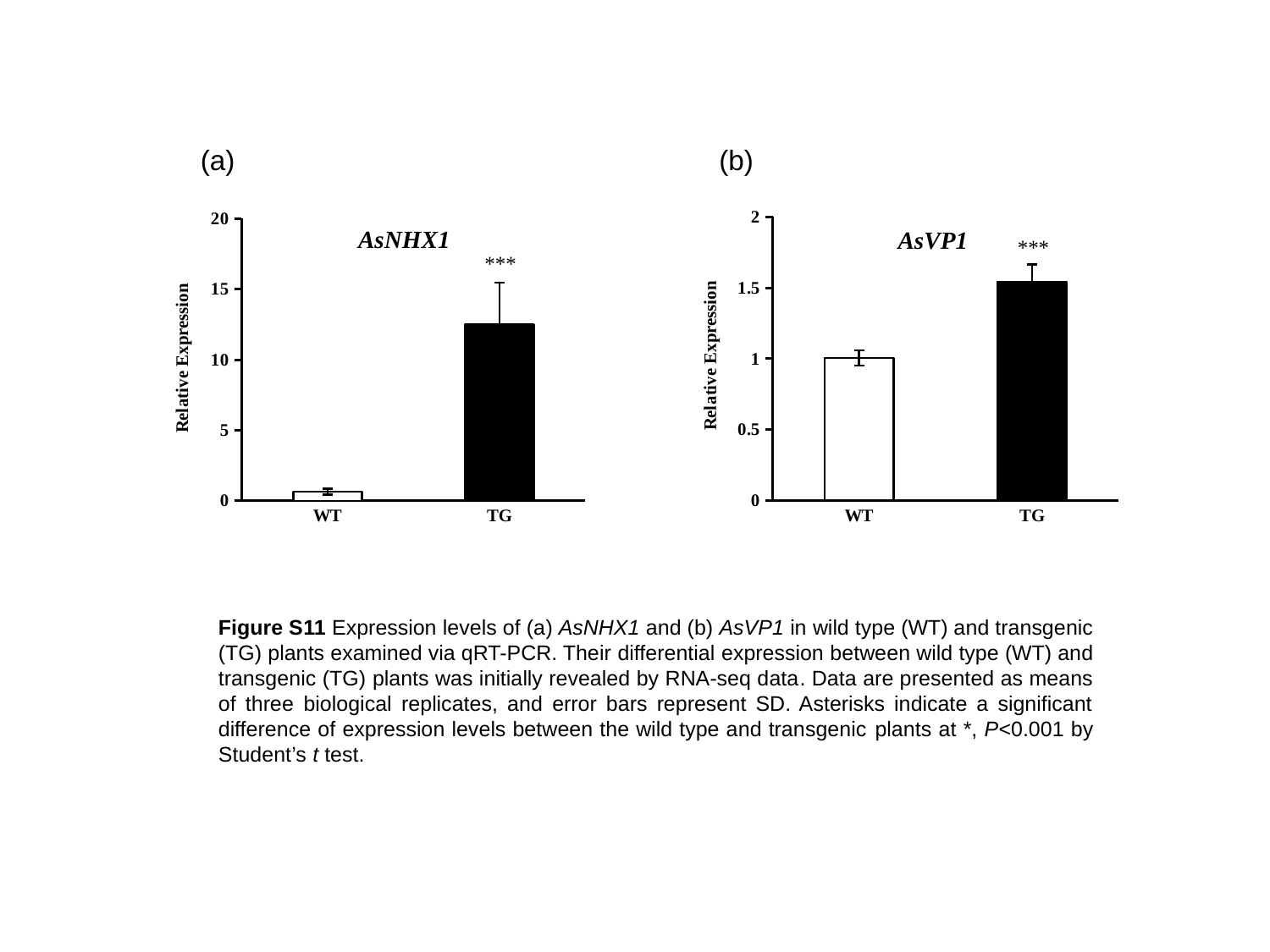

(a) (b)
### Chart
| Category | |
|---|---|
| WT | 1.004058761264813 |
| TG | 1.541601415843171 |AsVP1
***
### Chart
| Category | |
|---|---|
| WT | 0.651128453189563 |
| TG | 12.496380729632 |AsNHX1
***
Figure S11 Expression levels of (a) AsNHX1 and (b) AsVP1 in wild type (WT) and transgenic (TG) plants examined via qRT-PCR. Their differential expression between wild type (WT) and transgenic (TG) plants was initially revealed by RNA-seq data. Data are presented as means of three biological replicates, and error bars represent SD. Asterisks indicate a significant difference of expression levels between the wild type and transgenic plants at *, P<0.001 by Student’s t test.

## Slide 12
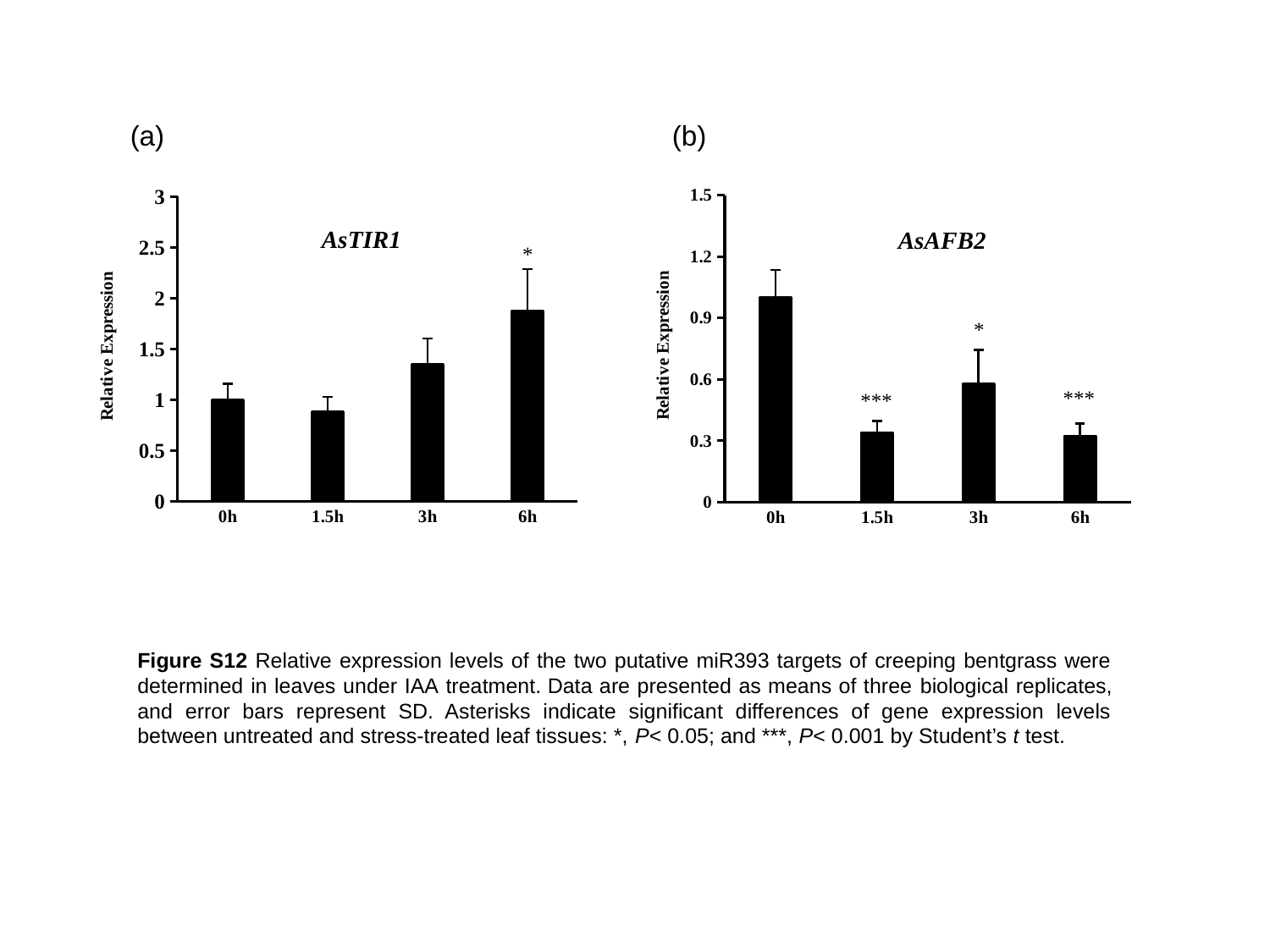

(a) (b)
### Chart
| Category | |
|---|---|
| 0h | 1.0 |
| 1.5h | 0.8849 |
| 3h | 1.3477 |
| 6h | 1.8788 |
### Chart
| Category | |
|---|---|
| 0h | 1.0 |
| 1.5h | 0.3402 |
| 3h | 0.579 |
| 6h | 0.3234 |AsTIR1
AsAFB2
*
*
***
***
Figure S12 Relative expression levels of the two putative miR393 targets of creeping bentgrass were determined in leaves under IAA treatment. Data are presented as means of three biological replicates, and error bars represent SD. Asterisks indicate significant differences of gene expression levels between untreated and stress-treated leaf tissues: *, P< 0.05; and ***, P< 0.001 by Student’s t test.
