# Supplementary material for: Transgenic creeping bentgrass overexpressing Osa‐miR393a exhibits altered plant development and improved multiple stress tolerance
Source: Plant Biotechnol J. 2018 Jul 4;17(1):233–51. doi: 10.1111/pbi.12960 (PMC6330543; doi:10.1111/pbi.12960)
Supplement: Supplementary file 2 — Table S1 Primer sequences used in this study. Table S2 The sequence of putative target genes for miR393 in creeping bentgrass, target sites are shown in red. [file PBI-17-233-s001.docx]

**Supporting information - Supplementary Tables**

**Table S1** Primer sequences used in this study.

| **Name** | **Sequence (5' - 3')** | **Purpose** |
| --- | --- | --- |
| ***Osa-miR393a* F** | TCTAGAGGTTTACAGTGTGATCATGGAG | Insert restriction site *XbaI* |
| ***Osa-miR393a* R** | GTCGACGAATAAAGAGCATGCATACCC | Insert restriction site *SalI* |
| ***Hyg* F** | TACACAGGCCATCGGTCCAGA | PCR for genomic DNA of transgenic plants |
| ***Hyg* R** | TAGGAGGGCGTGGATATGTC |  |
| ***miR393a* stem-loop RT** | GTCGTATCCAGTGCAGGGTCCGAGGTATTCGCACTGGATACGACGATCA | For reverse transcript of *miR393* |
| ***AsUBQ* q-F** | GGCGTCATCGACCTTGTAGA | For q-PCR of *AsUBQ* |
| ***AsUBQ* q-R** | GACAACGTCAAGGCCAAGAT |  |
| ***miR393* q-F** | GACTGTCCAAAGGGATCGCATT | For q-PCR of *miR393* |
| ***miR393* q-R** | GTGCAGGGTCCGAGGTATTC |  |
| ***AsTIR1* q-F** | AGAGGTCTTGTGGATGTTTCTG | q-PCR for mRNA of *AsTIR1* |
| ***AsTIR1* q-R** | ACGGTTCTTTGCGATCCTAAT |  |
| ***AsAFB2* q-F** | GACAGACGACTTCCAAACAGAG | q-PCR for mRNA of *AsAFB2* |
| ***AsAFB2* q-R** | GATGTAACGCAGAGAGCAGAAG |  |
| ***AsHSP17.0* q-F** | AAGGTGGAGGTCGAGGAT | q-PCR for mRNA of *AsHSP17.0* |
| ***AsHSP17.0* q-R** | TGAAGCGCCTGACAAACT |  |
| ***AsHSP26.7a* q-F** | CCCACAACACGTCGTTCA | q-PCR for mRNA of *AsHSP26.7a* |
| ***AsHSP26.7a* q-R** | CCGCATCTTCACCTCCTTATC |  |
| ***AsVP1* q-F** | TATGGTCTTGGCGGTTCTTC | q-PCR for mRNA of *AsVP1* |
| ***AsVP1* q-R** | GGTCATCTTCAGGGATGTTCTT |  |
| ***AsNHX1* q-F** | CATATACCTCCTGCCTCCAATC | q-PCR for mRNA of *AsNHX1* |
| ***AsNHX1* q-R** | GTCCCAACAGCACCAAATAATG |  |
| **DN219285_c3_g2_i3 q-F** | CTGGTTCTCAGTTTGTTCTGATTT | q-PCR for mRNA of DN219285_c3_g2_i3 |
| **DN219285_c3_g2_i3 q-R** | TTGTCTCGTGTATCGCATCC |  |
| **DN216241_c0_g3_i2 q-F** | GCGAGCAGGAGAACGAC | q-PCR for mRNA of DN216241_c0_g3_i2 |
| **DN216241_c0_g3_i2 q-R** | GACACCTACGCAAGATGGATAG |  |
| **DN216428_c0_g2_i1 q-F** | CCACCTCTAGCGATTCTTCTG | q-PCR for mRNA of DN216428_c0_g2_i1 |
| **DN216428_c0_g2_i1 q-R** | CATGTAGTACGAGCCCAAGTC |  |
| **DN223158_c1_g1_i1 q-F** | CCCACCACTCAGATAACACTTT | q-PCR for mRNA of DN223158_c1_g1_i1 |
| **DN223158_c1_g1_i1 q-R** | GCCACTCTCTCAAACCTATCTC |  |
| **DN224490_c0_g1_i1 q-F** | GTAGCGACTGTTGAGGAAGTC | q-PCR for mRNA of DN224490_c0_g1_i1 |
| **DN224490_c0_g1_i1 q-R** | ACTATGTGAGCACCAGCATAAA |  |
| **DN223637_c0_g2_i3 q-F** | GTCGTTAAGTCCGTGTCAGTAG | q-PCR for mRNA of DN223637_c0_g2_i3 |
| **DN223637_c0_g2_i3 q-R** | GTACATGGCAGTAGAGGGATTG |  |
| **DN218235_c1_g5_i2 q-F** | GCATCTCCTCCGACAAGTTC | q-PCR for mRNA of DN218235_c1_g5_i2 |
| **DN218235_c1_g5_i2 q-R** | ACAAGACTTTCTCAGCCATCTC |  |
| **DN212321_c0_g1_i3 q-F** | GCAGGATCTAACCAAGGAGAAG | q-PCR for mRNA of DN212321_c0_g1_i3 |
| **DN212321_c0_g1_i3 q-R** | TGGCAGACACACTTGTAATGA |  |
| **DN236376_c0_g1_i2 q-F** | GAGGACCAGGAGATGCTTATTT | q-PCR for mRNA of DN236376_c0_g1_i2 |
| **DN236376_c0_g1_i2 q-R** | CAGCTTCTTCAGGGTTGTAGT |  |

**Table S2** The sequence of putative target genes for *miR393* in creeping bentgrass, target sites are shown in red.

***AsTIR1* (AS28037):**

ATGGGCCGCGGCGGCCCCGCCGCGCCCCCGTGGCACTCGCTCCCGGACGAGGTCTGGGAGCACGCCTTCTCCTTCCTCCCCGCAGACGCCGACCGCGGCGTCGCGGCGGGCGTCTGCCACGGCTGGCTCCGCGCCGAGCGCCGCTCCCGGCGCCGCCTCACCGTCCCCAACTGCTACGCCACGGCCCCGCGCGACGCCGTCGAGAGGTTCCCCTCCGTCCGCGCCGCCGAGGTCAAGGGCAAGCCGCACTTCGCCGACTTCGGCCTCCTGCCGCCCTCCTGGGGCGCCTACGCCGCGCCCTGGGTCGCCGCCGCCGCCGACGGCTGGCCGCTACTCGAGGAGCTCAGCTTCAAGCGCATGTTCGTCACCGACGAGTGCCTCGAGATGATCGCGTCATCCTTCAGGAACTTCCAGGTCTTGCGCCTCGACTCCTGTGAGGGCTTCACCACCGCCGGCCTCGCCGCCATTACCGAAGGTTGCAGAAATTTAAGAGTATTTGACCTGCAAGAGAACTACATTGACGATTGTTCAAGTCACTGGCTCAGCAACTTTCCAGAAACCTTCACTTCTCTGGAAACTCTGAATTTTTCATGCTTAAACGGGGAGGTCAATTTCACTGTACTTGAGAGGCTAGTAAGCAGATGCCGCAACCTCAAGACTCTAAAGCTCAACAATGCTATCCCTCTTGACAATGTTGCTAGCCTACTTCATAAGGCTCCACAGATAATAGAACTCGGAACTGGAAAATTCTCTGCCGACTATCATCCAGATCTTTTCGCAAAGGTTGAAGCGGCATTTGCAGGTTGTAATAGCCTGAGAAGGCTTTCTGGGACATGGGATGCAGTTCCAGATTACCTGCCAGCATTCTATTGTGTTTGTGAAGGCCTCACATCTCTTAATCTGAGTTATGCCACTGTGCAAGGCCCTGAGCTCATAAAATTCATTAGCAGATGCAAGAATTTGCTGCAATTATGGGTGATGGACCTCATTGAAGACCATGGTCTGTCTGTTGTGGCATCAAGTTGCAGCAAACTGCAAGAGTTGCGGGTCTTTCCTTCTGATCCTTTTGGTCATAACGGCGGGCAAGTTTTCTTGACAGAAAGAGGTCTTGTGGATGTTTCTGCCAGTTGCCCCATGTTGGAGTCAGTTCTCTACTTCTGCAGCCGCATGACGAATGAGGCCCTTATTAGGATCGCAAAGAACCGTCCCAACTTCACTTGCTTCCGCTTAGCCCTCCTCGAGCCCCGTTCTCCGGATTACCTCACACGGCAGCCTCTTGATGCGGGTTTCAGTGCCATTGTGGAATCATGCAAGGGCCTTAGGCGTCTCTCCATGTCTGGTCTTCTCACAGATCTTGTGTTCAAATCAATAGGTGCACATGCTAATCGTCTTGAGATGCTCTCACTCGCCTTTGCTGGTGACAGCGATCTGGGCCTGAATGACATCCTCTCAGGCTGCAAGAGCCTGAAGAAGCTGGAGATCAGGGATTGCCCATTTGGGGATAAGGCGTTGCTGGCAAATGTGGCCAAGCTG**GAGACAATGCGATCCCTTTGG**ATGTCATCGTGCAATGTCACAGAGAAGGGGTGCCAAATCCTTGCGTCAAAGATGCCAATGCTTAATGTGGAGGTCATAAATGAGGTAGATGAAAGCAATGAAATGGATGAGAACCATGGAGGACTCCCCAAAGTGGACAAACTATATGTTTACCGAACAACTGCTGGGGCAAGGGATGATGCGCCAAATTTTGTTAAAATCCTTTAG

***AsAFB2-1* (AS37944):**

ATGACCTACTTTCCTGAGGAGGTGGTGGAGCACATATTCAGCTTCTTGCCTGCCCAATGCGACCGGAACAACGTTTCCCTTGTATGCAAGGTATGGTATGAGATTGAAAGGCTCAGCCGTCGAGATGTCTTTGTGGGGAACTGCTATGCCGTGCGCCCTGAGCGCGTGGTGCTTCGGTTCCCCAATGTGCGGGCACTGACAGTGAAGGGGAAGCCACACTTCGCTGACTTTAACCTTGTGCCACCTGATTGGGGTGGTTACGCTGGACCATGGATCGAGGCGGCGGCCAGGGGCTGTGTTGGTCTTGAGGAGCTTCGGATGAAGCGGATGGTGGTGTCCGATGAGAGCCTTGAGCTGCTTGCAAAATCATTCCCACGGTTCAAGGCCCTCATCCTTATCAGCTGTGAGGGGTTCAGCACCGATGGGCTAGCAGCTATTGCAAGTCACTGCAAGCACCTGAGGGAGTTAGATTTGCAGGAAAATGAAGTGGACGATCGAGGGCCAAGGTGGCTCTCCTGCTTCCCAGATTCATGCACATCCCTTGCCTCCTTGAACTTTGCCTGCATCAAAGGGGAGGTTAATGCTGGTTCATTGGAGAGACTTGTTGCTAGGTCCCCAAATCTCCGAAGTTTGAGGTTGAATCGATCTGTATCAGTAGATACACTCTCGAAGATATTATTGCGCACCCCTAATTTGGAGGACCTAGGGACTGGGAACTTGACAGACGACTTCCAAACAGAGTCCTATATCAGGCTGACCCTTGCTCTAGAGAAATGCAAAATGCTGAAGAGCTTGTCGGGCTTTTGGGATGCTTCTGCTCTCTGCGTTACATCCATCTATCCTGTTTGTGCACGACTAACAGGTTTAAACTTGAGCTATGCTCCTACTCTTGATTCTTCCGATCTCACCAAAATGATCAGTCACTGTGTGAAACTCCAACGTCTTTGGGTACTGGATTGCATTGCAGATAAGGGCTTGCAAGTGGTGGCCTCCAGTTGCAAGGATCTACAAGAACTAAGGGTATTTCCATCAGACTTCTATGTTGCTGGGTATTCCCCAGTGACGGAAGAGGGACTTGTTGCAATTTCCATGGGCTGTCCAAAATTGAGCTCATTGCTGTATTTCTGTCATCAAATGACCAATGCTGCACTAATTGCTATTGCTAAGAACTGCCCAAATTTCACACGGTTCAGACTCTGTATTCTTGAGCCTGGGAAGCCTGATGCCATGACGAACGAACCACTAGATGAAGGTTTTGGTGCTATTGTTCGTGAATGCGAAGGGCTCAGGCGACTGTCAATATCGGGTCTTCTTACTGACAAGGTTTTCATGCATATTGGGAGATATGCAAAACAACTTGAGATGCTTTCAATAGCATTTGCTGGAGATAGTGATGCAGGTATGATGCATGTTATGGAAGGATGCAAGAATCTAAGGAAGCTGGAGATTAGAGATAGCCCATTTGGTGATGCTGCACTCTTGGAGAATGTTGCCAAGTAT**GAGACAATGCGATCCCTTT**GGATGAACTCGTGCTTGTTGACCGTGGGCGCATGCCGACTGCTTGCACTCAAGATGCCTCACCTCACTGTGGAGATAATAAACGATCCTGGAGAGACATGCCCGGTGGAGTCACTTCCATTTGATAGCCCTGTTGAGAAACTGTATGTCTACCGGACACTTGCGGGTCCAAGGTCTGACACACCAGACTGCGTCCAGATTGTTTAG

***AsAFB2-2* (AS37945):**

ATGACCTACTTTCCTGAGGAGGTGGTGGAGCACATATTCAGCTTCTTGCCCGCCCAATGTGACCGGAACACGGTTTCGCTTGTATGCAAGGTATGGTATGAGATTGAAAGGCTCAGCCGTCGAGATGTCTTTGTGGGGAACTGCTATGCCGTGCGCCCTGAGCGCGTGGTGCTTCGGTTCCCCAATGTGCGGGCGTTGACGGTGAAGGGGAAGCCTCACTTTGCTGACTTCAACCTTGTGCCACCTGATTGGGGTGGCTATGCTGGACCATGGATCGAGGCGGCGGCCAGGGGCTGTGTGGGTCTTGAGGAGCTGCGGATGAAGCGGATGGTGGTGTCGGATGAGAGCCTTGAGCTGCTTGCGAAATCATTCCCACGATTCAAGGCCCTCATCCTTATCAGCTGTGAGGGGTTCAGCACCGATGGGCTAGCAGCTATTGCAAGTCACTGCAAGCTCCTGAGGGAGTTGGATTTGCAGGAAAATGAAGTAGACGATCGAGGGCCAAGGTGGCTCTCCTGCTTCCCAGATTCCTGCACAACCCTTGCCTCCTTGAATTTTGCCTGCATCAAAGGGGAGGTTAATGCTGGTTCATTGGAGAGACTTGTTGCTAGGTCTCCAAATCTTCGAAGTTTGAGGTTGAATCGATCTGTATCAGTAGACACACTCTCGAAGATACTAATGCGCACCCCTAATTTGGAGGACCTAGGAACTGGGAACTTGACAGATGACTTCCAAACTGAGTCCTATCTCAGGCTGACCCAAGCTCTAGAGAAATGCAAAATGTTGAAGAGCTTGTCGGGCTTTTGGGATGCTTCTGCTCTCTGCGTTCCATTCGTCTATCCTGTCTGTGCGCAACTAACAGGTTTAAACTTGAGCTATGCTCCTACACTTGATTCCTCCGATCTCACCAAAATGATCAGTCACTGTGTGAAACTCCAACGTCTTTGGGTACTGGATTGCATTGCAGATAAGGGCTTGCAAGTGGTGGCCTCCAGTTGCAAGGATCTACAAGAACTTAGGGTATTCCCATCAGACTTCTATGTTGCTGGGTATTCCCCCGTGACAGAAGAGGGGCTTGTTGCAATATCCAGGGGCTGTCCAAAATTGAGCTCATTGCTGTATTTCTGTCATCAAATGACCAATGCTGCACTAATGACTATTGCTAAGAACTGCCCAAATTTCACGCGGTTTAGACTCTGTATTCTTGAGCCTGGGAAGCCTGATGCCTTGACAAACCAACCATTAGATGAAGGTTTTGGTGCTATTGTTCGTGAATGCGAAGGGCTCAGGCGACTGTCAATATCGGGTCTTCTTACTGACAAGGTTTTCATGCATATTGGGAGATATGCAAAACAACTTGAGATGCTTTCAATAGCATTTGCTGGAGATAGTGATGCAGGAATGATGCATGTTATGGGAGGATGCAAGAATCTAAGGAAGCTGGAGATTAGAGATAGCCCATTTGGTGATGCTGCACTCTTGGAGAATGTTGCCAAGTAT**GAGACAATGCGATCCCTTT**GGATGAACTCGTGCTTGTTGACCGTGGGCGCATGCCGACTGCTTGCACTCAAGATGCCTCACCTCACTGTGGAGATAATAAACGATCCTGGAGAAACATGTCCGGTGGAGTCACTTCCGTTTGATAGCCCTGTTGAGAAATTGTATGTCTACCGGACACTTGCCGGTCCAAGGTCCGACACACCAGACTGCGTCCAGATTGTTTAG
